# Integrated analysis highlights APC11 protein expression as a likely new independent predictive marker for colorectal cancer

**DOI:** 10.1038/s41598-018-25631-1

**Published:** 2018-05-09

**Authors:** Youenn Drouet, Isabelle Treilleux, Alain Viari, Sophie Léon, Mojgan Devouassoux-Shisheboran, Nicolas Voirin, Christelle de la Fouchardière, Brigitte Manship, Alain Puisieux, Christine Lasset, Caroline Moyret-Lalle

**Affiliations:** 1 Centre Léon Bérard, Département de Santé Publique, Lyon, F-69008 France; 2 CNRS UMR 5558, Laboratoire de Biométrie et Biologie Evolutive, Lyon, F-69373 France; 3 Centre Léon Bérard, Service d’Anatomopathologie, Lyon, F-69008 France; 4 INRIA Grenoble-Rhône-Alpes, 655 Avenue de l’Europe, 38330 Montbonnot, Saint Martin France; 5 Synergie Lyon Cancer, Plateforme de Bioinformatique ‘Gilles Thomas’ Centre Léon Bérard, Lyon, France; 6 Centre Léon Bérard, Lyon, F-69008 France; 7 INSERM U1052, Cancer Research Center of Lyon, Lyon, F-69008 France; 8 CNRS UMR 5286, Cancer Research Center of Lyon, Lyon, F-69008 France; 9 Université de Lyon, Lyon, F-69622 France; 10 Université Lyon1, ISPB, Lyon, F-69008 France; 11 LabEx DEVweCAN, Université de Lyon, F-69000 Lyon, France; 12 Hôpital de la Croix Rousse, Hospices Civils de Lyon, Lyon, F-69008 France; 13 Hospices Civils de Lyon, Hôpital Edouard Herriot, Service d’Hygiéne, Epidémiologie et Prévention, Lyon, F-69437 France

## Abstract

After a diagnosis of colorectal cancer (CRC), approximately 50% of patients will present distant metastasis. Although significant progress has been made in treatments, most of them will die from the disease. We investigated the predictive and prognostic potential of APC11, the catalytic subunit of APC/C, which has never been examined in the context of CRC. The expression of APC11 was assessed in CRC cell lines, in tissue microarrays (TMAs) and in public datasets. Overexpression of APC11 mRNA was associated with chromosomal instability, lymphovascular invasion and residual tumor. Regression models accounting for the effects of well-known protein markers highlighted association of APC11 protein expression with residual tumor (odds ratio: OR = 6.51; 95% confidence intervals: CI = 1.54–27.59; P = 0.012) and metastasis at diagnosis (OR = 3.87; 95% CI = 1.20–2.45; P = 0.024). Overexpression of APC11 protein was also associated with worse distant relapse-free survival (hazard ratio: HR = 2.60; 95% CI = 1.26–5.37; P = 0.01) and worse overall survival (HR = 2.69; 95% CI = 1.31–5.51; P = 0.007). APC11 overexpression in primary CRC thus represents a potentially novel theranostic marker of metastatic CRC.

## Introduction

Colorectal cancer (CRC) is the third most frequent cancer and the fourth cause of cancer-related mortality worldwide^1^. Patient survival is highly dependent on the stage of CRC at the time of diagnosis but approximately 50% of the patients will be concerned by distant metastasis development, either present at diagnosis (20%) or occurring after the curative-intent surgery of the primary tumor. The most frequent sites affected by metastatic CRC (mCRC) are the liver and lung^1^. The current first-line standard-of-care for mCRC relies on the combination of cytotoxic chemotherapy (5FU/FA, oxaliplatin, irinotecan) and biologic agents (anti VEGF(R) or anti-EGFR monoclonal antibodies) guided by the molecular profile of the tumor. Surgery or local tumor ablation may also play a role in the treatment of mCRC patients, especially those with oligometastatic disease.

Several biomarkers, mostly predictive, are routinely used for mCRC^2,3^. Activating *RAS* mutations (*KRAS* and *NRAS*), present in nearly 50% of mCRC cases, are negative predictive markers of anti-EGFR inhibitor efficacy (cetuximab, panitumumab) and RAS testing is now mandatory in all mCRC patients, from the first-line metastatic setting. V600E-*BRAF* mutation is a significant negative poor-prognostic marker for patients with mCRC and may be a negative predictive factor for anti-EGFR therapies.

Biomarkers of chemotherapy sensitivity and toxicity including DPD (Dihydro Pyrimidine Dehydrogenase) and UGT1A1 (UDP-Glucuronosyl Transferase 1A1) are optionally evaluated in the management of patients with mCRC^4^. Other biomarkers like APC or TP53 are not routinely used for a prognostic or therapeutic purpose in mCRC^5,6^. Immune checkpoint (PD1-PDL1) inhibitors have also given rise to interesting results in high level microsatellite instability (MSI-H) mCRC patients^7,8^. Despite these improvements, the median overall survival for mCRC patients is limited, reaching 30 months in hyperselected patients^9–11^.

More recently, a molecular re-classification of CRCs, namely the consensus molecular subtypes (CMS), has been developed by Guinney and colleagues^12^. Of these new categories, CMS1 appears to represent the previously designated microsatellite-instable (MSI) subtype, while the “canonical” (CMS2) and “mesenchymal” (CMS4) subtypes most likely encompass the previously described chromosomal instability (CIN) or microsatellite-stable (MSS) subtypes. A final category, the “metabolic” (CMS3), shows a disruption of metabolic pathways that contains *KRAS* activating mutations known to induce metabolic adaptation. The CMS2 and CMS4 subtypes interestingly display elevated somatic copy number alterations (SCNA) and cell cycle mRNA gene set over enrichment for the CMS2, similar to the MSS subtype characterized by aneuploidy, multiple chromosomal rearrangements and an accumulation of somatic mutations^12^.

**Figure 1:**
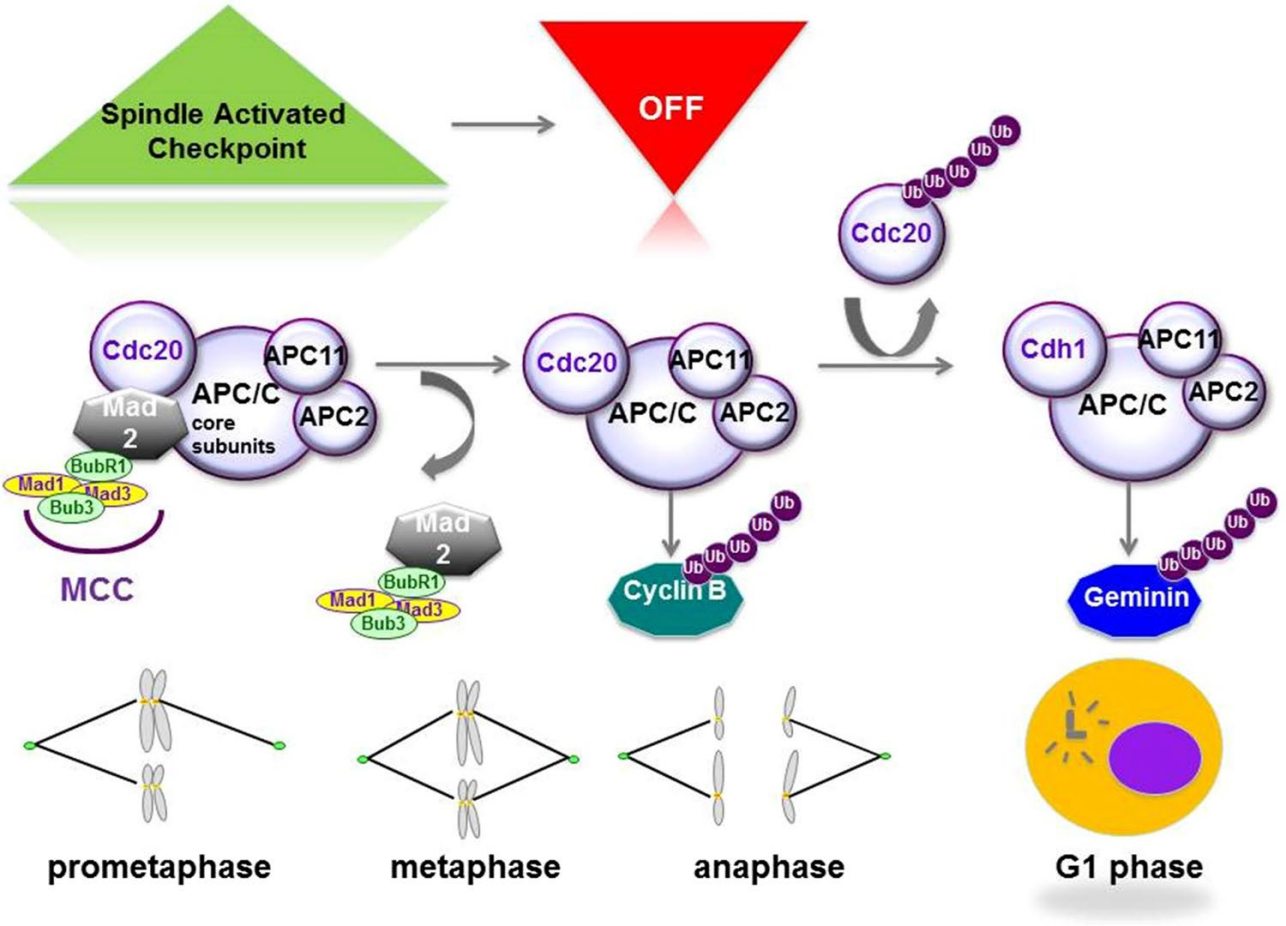
Regulation and function of APC/C. The activator of APC/C, CDC20 (cell division cycle 20) is inhibited by MCC (Mad2, BubR1, Mad1, Mad3) sequestration until all of the spindles have attached to kinetochores at metaphase; this system is referred to as the SAC (spindle activated checkpoint). Once all of the chromosomes are bi-orientated on a metaphase plate, the SAC is extinguished. Release from SAC activates APC/CCDC20. APC2/APC11 catalytic E3 sub-complex activity promotes proteolysis by poly-ubiquitination of APC/C targets, such as cyclin B, leading to anaphase onset. APC/C then switches its activator from CDC20 to Cdh1. The newly formed APC/CCdh1 complex drives mitotic exit by targeting CDC20 for destruction. During the G1 phase, APC/CCdh1 targets several regulators of DNA replication, such as Geminin. After the degradation of its substrates in G1, APC/C catalyzes the auto-ubiquitination of the APC11 subunit, which confers E3 activity, and its E2 ubiquitin-conjugating enzyme UbcH10, leading to APC/CCdh1 inactivation.

It is now admitted that these chromosomal instabilities and ensuing CIN+/SCNA+ tumors may arise from dysregulated cell cycle mechanisms, such as the proteolysis of key cell cycle elements (mitotic oscillators)^13,14^. Indeed, cell division, including mitosis, is governed by the degradation of different regulatory proteins by ubiquitin-dependent proteolysis. The anaphase-promoting complex/cyclosome (APC/C) is a specific E3 ubiquitin ligase complex that is essential for chromosome segregation, exit from mitosis, and the subsequent stable isolation of the G1 phase to control entry into the S phase (Fig. 1)^15^. More recently, APC/C has been involved in regulation of genomic integrity, apoptosis, metabolism, neurodifferentiation and development through degradation of specific proteins^16,17^. Mitotic cyclins and securin are key proteosomal targets of APC/C and known to be dysregulated in cancer. Abnormal expression of cyclin B1 or securin is considered to be a major factor in the development of polyploidy^18,19^. APC/C is a large multiprotein E3 ligase complex which consists of three sub-complexes^16^: the catalytic one contains APC2, APC10 and the RING-H2 finger protein APC11, the scafolding subcomplex platform corresponds to APC1, APC4 and APC5 subunits and a tetratricopeptide repeat (TPR) arm composed of APC3, APC6, APC7 and APC8 subunits, interacting with one of the two co-activators (Cdc20 or Cdh1). On the platform, the APC1 subunit represents the bridge between the catalytic portion and the TPR arm. The activity of the complex and recruitment of substrates are dependent on the co-activator subunit. Mechanistically, it was shown that a heterodimeric complex of APC2 and APC11 is sufficient to catalyze the ubiquitination of human securin and cyclin B1^20^ with APC11 regulating the interface with E2 enzymes^21^. The activity of the APC/C complex is repressed by the mitotic checkpoint complex (MCC) composed of at least four subunits, including Mad2, Bub3 and BubR1 which sequesters the Cdc20 subunit and induces the spindle activated chekpoint (SAC)^22^. Alterations in MAD2, BUBR1 and BUB1 expression were reported in cancer, and inherited mutations in the BUBR1 gene are proposed to cause CIN and to predispose to cancer^23^. After long considering the SAC as a safeguarding mechanism, it is now admitted that a sustained SAC may lead alternatively to cell death or aneuploidy. A better understanding of the co-activator function stressed the Janus face of APC/C in cancer with Cdc20 playing a pro-tumor role in numerous cancer types including CRC^24^, while Cdh1 is now considered as a tumor suppressor^25,26^. To date, very few studies have reported alterations in the APC/C core subunits in cancer^26–28^. Our group was the first to determine the gene status of nine APC/C subunits (*APC2*, *APC3*, *APC4*, *APC5*, *APC6*, *APC7*, *APC8*, *APC10* and *APC11*) in cancer cells of different human tissue origins, and identified the presence of several heterozygote mutations in platform and TPR arm subunits genes (*APC4*, *APC6* and *APC8*) in cell lines of CRC origin^25^. We have shown that the transient expression of a truncated mutant of APC8 subunit leads to abnormal levels of APC/C targets such as cyclin B1 and disturbs the cell cycle progression of colon epithelial cells through mitosis^25^. Further investigation of the APC/C subunits mRNA expression revealed a significant association of APC11 expression with the CRC cell lines (data not shown). The APC11 subunit appears of particular interest since it plays a central role in binding to the acceptor ubiquitin and enhances diubiquitin formation by the E2 protein Ube2S^21^. Very recently, Sansregret *et al*.^28^ have reported a total of 132 missense mutations in APC/C subunits in cancer with the vast majority of them (93) affecting APC/C structure’s subunits while only 3 out of 132 occurred in the *APC11* gene. Moreover, in contrast to the other catalytic core subunit APC2, which was shown to be down-regulated in various cancer types, APC11 expression has never been investigated previously in cancer.

In the current study, based on different mRNA and protein expression analyses (RT-qPCR, western blot, TMA of 82 primary colorectal cancer tissues, CCLE^29^ and TCGA^30^ datasets), we aimed at delineating the involvement of APC11 expression in CRC tumorigenesis. Overexpression of APC11 is significantly correlated with chromosomal instability (while no association was found for the expression of the other catalytic subunits APC2 and APC10). A significant association is observed between APC11 expression and lymphovascular invasion and residual tumor. High levels of APC11 protein in primary colorectal tumors is specifically correlated with metastasis at diagnosis. Using multifactorial analyses and multivariable regression models we also show, that alongside well-known markers involved in CRC tumorigenesis, namely Ki67, p53, E-cadherin, Bcl2, MLH1, MSH2, and DCC^26^, APC11 appears as an independent and potentially important new predictive factor.

## Results

### APC11 expression in CRC cell lines

#### mRNA expression

**Figure 2:**
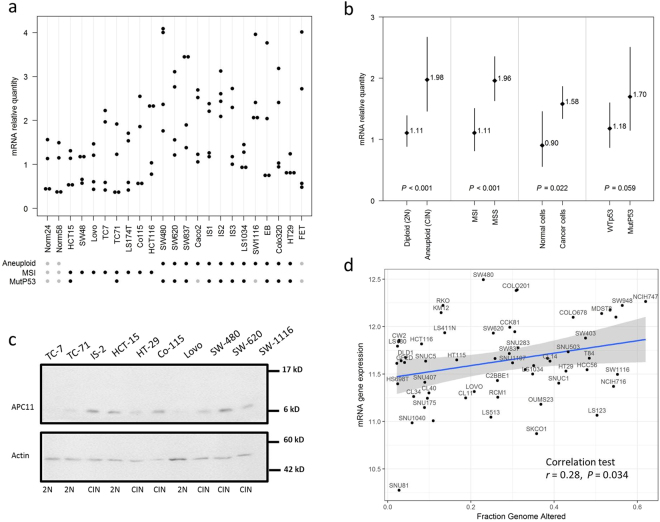
APC11 expression in CRC cell lines and statistical correlations with clinical and biological features. (**a**) Relative levels of *APC11* mRNA were measured using real-time RT-PCR. The ratios of APC11/reference genes of four independent RT-PCR are depicted individually in the figure. Individual cell lines genetic status is indicated by dots at the bottom of the panel, grey dots indicate missing data. (**b**) The results of the statistical analysis of the individual data presented in panel (a) are displayed using a graphical representation. For each case, the number indicates the mean expression and the bars the 95% confidence intervals estimated by a random effects model. (**c**) Western blot analysis in colon cancer cell lines. The signal intensity of APC11 was normalized against actin. In the figure, are reported the cropped gel/blots for each protein evaluation. The black boxed indicate the cropped regions. Uncropped full-length gel/blots are presented in Fig. S2 (see Supplemental information). Relative protein expression was estimated using the Quantity One software (BioRad, Marnes-la-Coquette, France). (**d**) *APC11* mRNA expression in 59 CRC cell lines from CCLE according to the fraction of genome altered (FGA) calculated with a threshold value of 0.3. The coefficient of correlation r is displayed with the corresponding *P* value; The regression line from a linear model (blue line) and its 95% confidence interval (grey area) are also displayed. Abbreviations: MSS, microsatellite stable; MSI, microsatellite instable.

We assessed the expression of *APC11*, the catalytic subunit of APC/C, in 21 CRC cell lines by RT-qPCR. We found a significant association between *APC11* mRNA expression levels and ploidy/microsatellite status (Fig. 2a,b). Indeed, a significantly higher mean *APC11* mRNA expression was observed in aneuploid compared to diploid and near diploid colon cancer cell lines (CIN: mean mRNA = 1.904; 95% CI = 1.386–2.616; vs. Diploid: mean mRNA = 0.999; 95% CI = 0.774–1.291; *P* for comparison <0.001), and MSI cell lines exhibited a lower mean level of *APC11* (MSI: mean mRNA = 1.11; 95% CI = 0.817–1.508; vs. MSS: mean mRNA = 1.962; 95% CI = 1.635–2.355; *P* for comparison <0.001). Mean levels of *APC11* mRNA in cancer cells were also higher than in immortalized epithelial cells (normal cells: mean mRNA = 0.904; 95% CI = 0.561–1.458; vs. cancer cells: mean mRNA = 1.583; 95% CI = 1.344–1.864; *P* for comparison = 0.022). Though not statistically significant, the expression of *APC11* also appeared to be associated with *TP53* gene status with mutated *TP53* cell lines exhibiting higher mean levels of *APC11* mRNA than *TP53* WT (mutated *TP53*: mean mRNA = 1.70; 95% CI = 1.152–2.507; vs. *TP53* WT: mean mRNA = 1.18; 95% CI = 0.872–1.60; *P* for comparison = 0.059). Similar observations were made in the Cancer Cell Line Encyclopedia (CCLE) dataset^29^ (Fig. 2d), where a significant correlation was found between *APC11* mRNA expression and the FGA status (Fraction Genome Altered) in 59 CRC cell lines (r = 0.28, *P* = 0.034). No correlation with the FGA status was observed for the other catalytic subunits APC2 and APC10 (Supplemental Fig. S1). Akin results were obtained when restricting the analysis to the subset of the 42 *TP53* wild-type-expressing CRC cell lines (data not shown). Moreover, no correlation was found between *TP53* mutational status and *APC11* mRNA expression in CCLE dataset (Supplemental Fig. S4a).

#### Protein expression

Semi-quantitative Western blot analysis of APC11 protein levels in colon cancer cell lines revealed a 60% concordance between mRNA and protein levels (Figs 2c and S2). Four out of the 6 aneuploid colon cancer cell lines displayed high levels of APC11 protein, while 3 out of the 4 diploid cell lines showed lower levels of APC11 protein, comparable to levels found in the immortalized epithelial cell line HME-1 (Figs 2c and S2).

Thus, APC11 expression is associated with chromosomal instability in CRC cell lines.

### APC11 expression in primary colorectal tumors

#### mRNA expression from TCGA datasets repository

**Figure 3:**
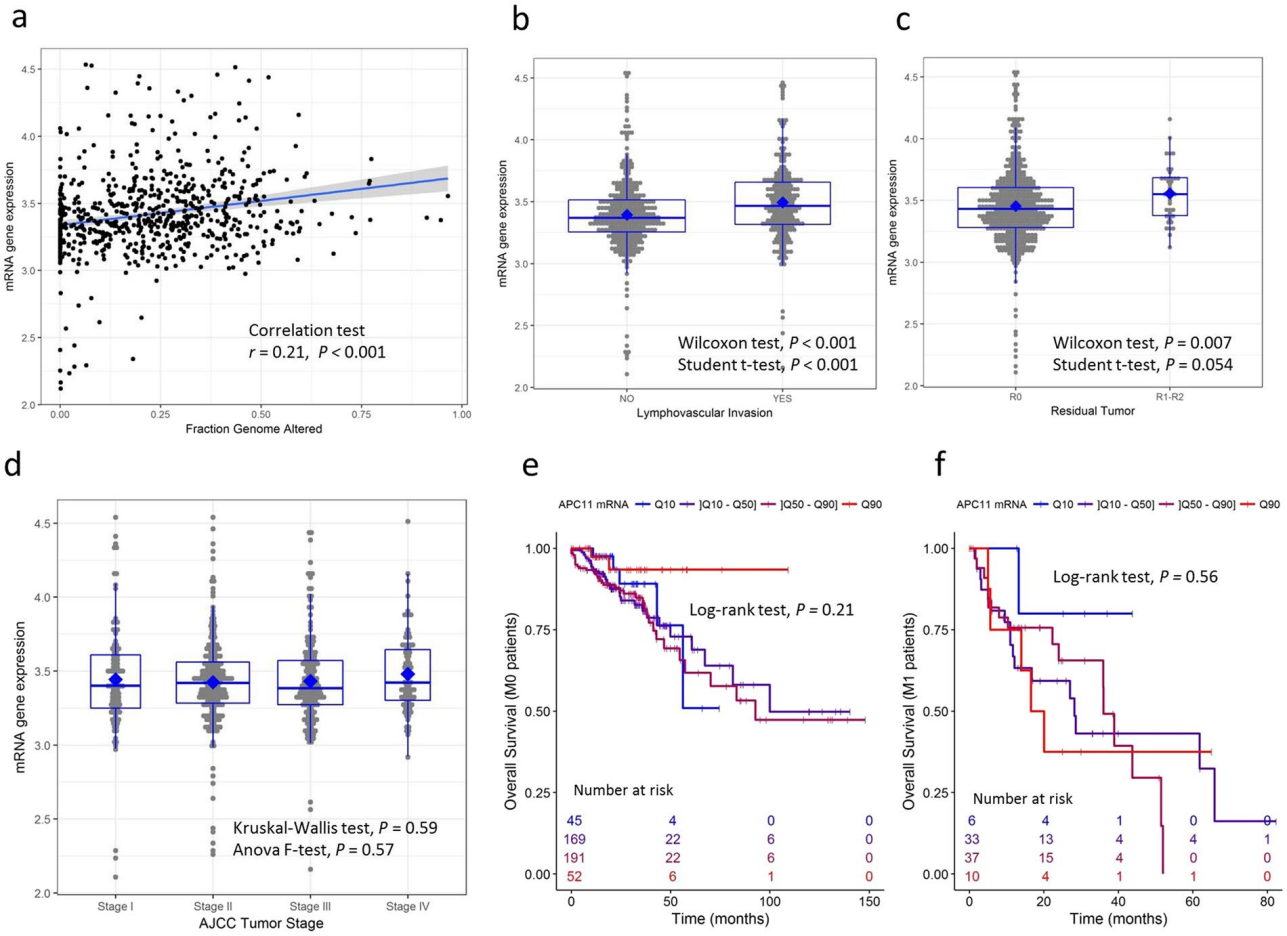
*APC11* mRNA expression in primary CRC from the TCGA repository and statistical correlations with clinical and biological features. Data of the TCGA READ cohort (rectum adenocarcinoma, N = 174) and the TCGA COAD cohort (colon adenocarcinoma, N = 499) were combined. (**a**) *APC11* mRNA expression according to the fraction of genome altered (FGA) calculated with a threshold value of 0.2. The coefficient of correlation *r* is displayed with the corresponding *P* value. The regression line from a linear model (blue line) and its 95% confidence interval (grey area) are also displayed. Panels (**b**–**d**) show respectively the *APC11* mRNA expression according to lymphovascular invasion, residual tumor status, and AJCC tumor stage. Panels (**e**–**f**) show the Kaplan-Meier curves of overall survival according to *APC11* mRNA expression stratified using quantiles, for patients with M0 disease (N = 457, panel e) and patients with M1 disease (N = 86, panel f).

A significant correlation between high expression of *APC11* mRNA and high levels of FGA was observed in the primary CRC datasets from TCGA repository (r = 0.21, *P* < 0.001, Fig. 3a). The expression of *APC10* mRNA was not significantly correlated with the FGA status, while decreased *APC2* mRNA levels appeared associated with the extent of FGA, though not notably (APC10: r = −0.01, *P* = 0.73; APC2: r = −0.07, *P* = 0.076, Supplemental Fig. S3). Similar results were obtained when restricting the analysis to the subset of 506 samples harboring a *TP53* wild-type gene status (data not shown). Moreover, no correlation was found between *TP53* mutational status and *APC11* mRNA expression in TCGA dataset (Supplemental Fig. S4b).

#### Clinical significance of mRNA expression

In TCGA CRC datasets, *APC11* mRNA elevated levels were significantly associated with lymphovascular invasion (*P* < 0.001, Fig. 3b) and with residual disease (*P* = 0.0071, Fig. 3c) but not with AJCC tumor stages (Fig. 3d). Overall survival analyses did not show any significance for *APC11* mRNA expression as a predictive marker for patients with M0 or M1 disease (Fig. 3e,f). Similar analyses were performed for APC10 and APC2 subunits. *APC2* and *APC10* mRNA levels were found associated with lymphovascular invasion, but no correlation was observed with residual disease, AJCC tumor stage, and overall survival, either for APC10 or APC2 (Supplemental Figs S5 and S6).

#### Protein expression in a series of 82 patients with primary CRC

**Figure 4:**
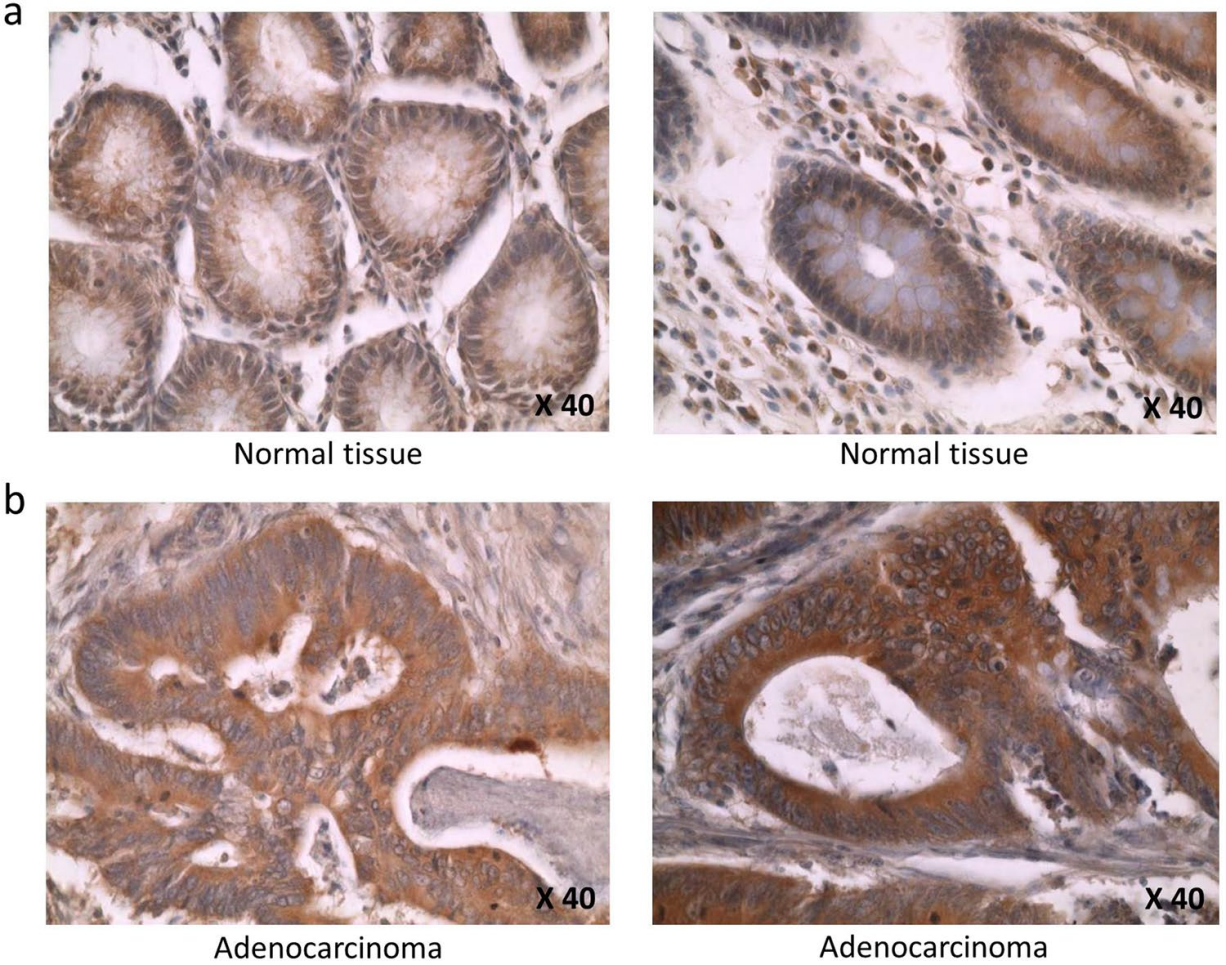
Immunohistochemical analysis of APC11 protein expression in normal and colorectal cancer tissues. (**a**) APC11 staining is restricted to the cytoplasm of epithelial normal colon cells. (**b**) High APC11-intensity staining is seen in the cytoplasm of colorectal cancer cells. The images are shown at 40X magnification.

The expression of the E3 ligase catalytic subunit APC11 protein was investigated using TMA consisting of 486 samples, including 191 pairs of cancer samples and corresponding normal mucosa^26^. Owing to a lack of clear staining of certain samples, only 82 tumor samples were deemed reliable and were analyzed. Examples of IHC results are shown in Fig. 4. Analysis of normal mucosa tissues revealed a uniform expression of the APC11 protein in the cytoplasm of luminal cells (corresponding to 50% stained cells) and a heterogeneous expression in myoepithelial or in stromal cells (Fig. 4a). In CRC cells, APC11 showed variable expression patterns; some tumors were negative for APC11 staining, others displayed a lower percentage of labeled cells (<50% stained cells), whereas others yet showed a higher percentage of stained cells than normal tissues (>50% stained cells) (Fig. 4b).

Hence, expression of the APC11 protein appears to be dysregulated in this cohort of primary colorectal tumors.

#### Clinical significance of protein expression

Baseline characteristics of the series of 82 patients stratified according to their APC11 expression level are given in Tables 1 and 2. Mean age at CRC diagnosis was 62 years, with approximately half of the patients diagnosed with nodal involvement and one third with metastases. As expected, no apparent selection bias was found by comparing the 82 and 109 patients who could and could not be characterized based on APC11 tumor expression (Table S1).

**Table 1:** Clinical and histopathological characteristics at diagnosis of our series of 82 patients with colorectal cancer, stratified by APC11 expression level.

Characteristics	N^a^	APC11 protein expression level		All tumors (N = 82)
		Marked cells ≤50% (N = 34)	Marked cells >50% (N = 48)	
Age at diagnosis (years)	82			
Mean ± SD		61.46 ± 12.7	63.37 ± 13.39	62.58 ± 13.06
[Min.–Max.]		[33.38–84.05]	[29.41–96.74]	[29.41–96.74]
Sex	82			
Male		19 (56%)	22 (46%)	41 (50%)
Female		15 (44%)	26 (54%)	41 (50%)
Stage TNM	82			
I		9 (26%)	5 (10%)	14 (17%)
II		12 (35%)	14 (29%)	26 (32%)
III		7 (21%)	7 (15%)	14 (17%)
IV		6 (18%)	22 (46%)	28 (34%)
Stage pTT4	82			
T1		6 (18%)	1 (2%)	7 (9%)
T2		5 (15%)	5 (10%)	10 (12%)
T3		21 (62%)	32 (67%)	53 (65%)
T4		2 (6%)	10 (21%)	12 (15%)
Node involvement pN	81			
N0		22 (65%)	20 (43%)	42 (52%)
N1		5 (15%)	12 (26%)	17 (21%)
N2		7 (21%)	15 (32%)	22 (27%)
Metastasis pM	82			
M0		28 (82%)	26 (54%)	54 (66%)
M+		6 (18%)	22 (46%)	28 (34%)
Tumor residue	82			
R0		31 (91%)	29 (60%)	60 (73%)
R1 and R2		3 (9%)	19 (40%)	22 (27%)
Tumour location	81			
Left colon and up rectum		21 (62%)	31 (66%)	52 (64%)
Right and transverse colon		13 (38%)	16 (34%)	29 (36%)
Differentiation	82			
Good and moderate		26 (76%)	30 (62%)	56 (68%)
Poor		8 (24%)	18 (38%)	26 (32%)
Vascular invasion	80			
Absence		28 (82%)	25 (54%)	53 (66%)
Presence		6 (18%)	21 (46%)	27 (34%)
Stroma	68			
Lymphoid		14 (50%)	28 (70%)	42 (62%)
Not lymphoid		14 (50%)	12 (30%)	26 (38%)
Ploidy	70			
Diploid		10 (34%)	13 (32%)	23 (33%)
Aneuploid		19 (66%)	28 (68%)	47 (67%)
Pre-operative CEA	66			
Normal		20 (74%)	19 (49%)	39 (59%)
Increased		7 (26%)	20 (51%)	27 (41%)

^a^Number of patients with available data.

**Table 2:** Immunohistochemistry characterization of the 82 colorectal cancer tissues, stratified by APC11 protein expression level.

Protein markers	N^a^	APC11 protein expression level		All tumors (N = 82)
		Marked cells ≤50% (N = 34)	Marked cells >50% (N = 48)	
E-cadherin	80			
−		9 (27%)	6 (13%)	15 (19%)
+		24 (73%)	41 (87%)	65 (81%)
KI67	81			
−		12 (36%)	32 (67%)	44 (54%)
+		21 (64%)	16 (33%)	37 (46%)
MLH1	78			
−		17 (52%)	22 (49%)	39 (50%)
+		16 (48%)	23 (51%)	39 (50%)
MSH2	76			
−		20 (67%)	30 (65%)	50 (66%)
+		10 (33%)	16 (35%)	26 (34%)
DCC	81			
−		15 (44%)	22 (47%)	37 (46%)
+		19 (56%)	25 (53%)	44 (54%)
P53	79			
−		15 (47%)	20 (43%)	35 (44%)
+		17 (53%)	27 (57%)	44 (56%)
BCL2	78			
−		21 (66%)	32 (70%)	53 (68%)
+		11 (34%)	14 (30%)	25 (32%)

^a^Number of patients with available data.

**Figure 5:**
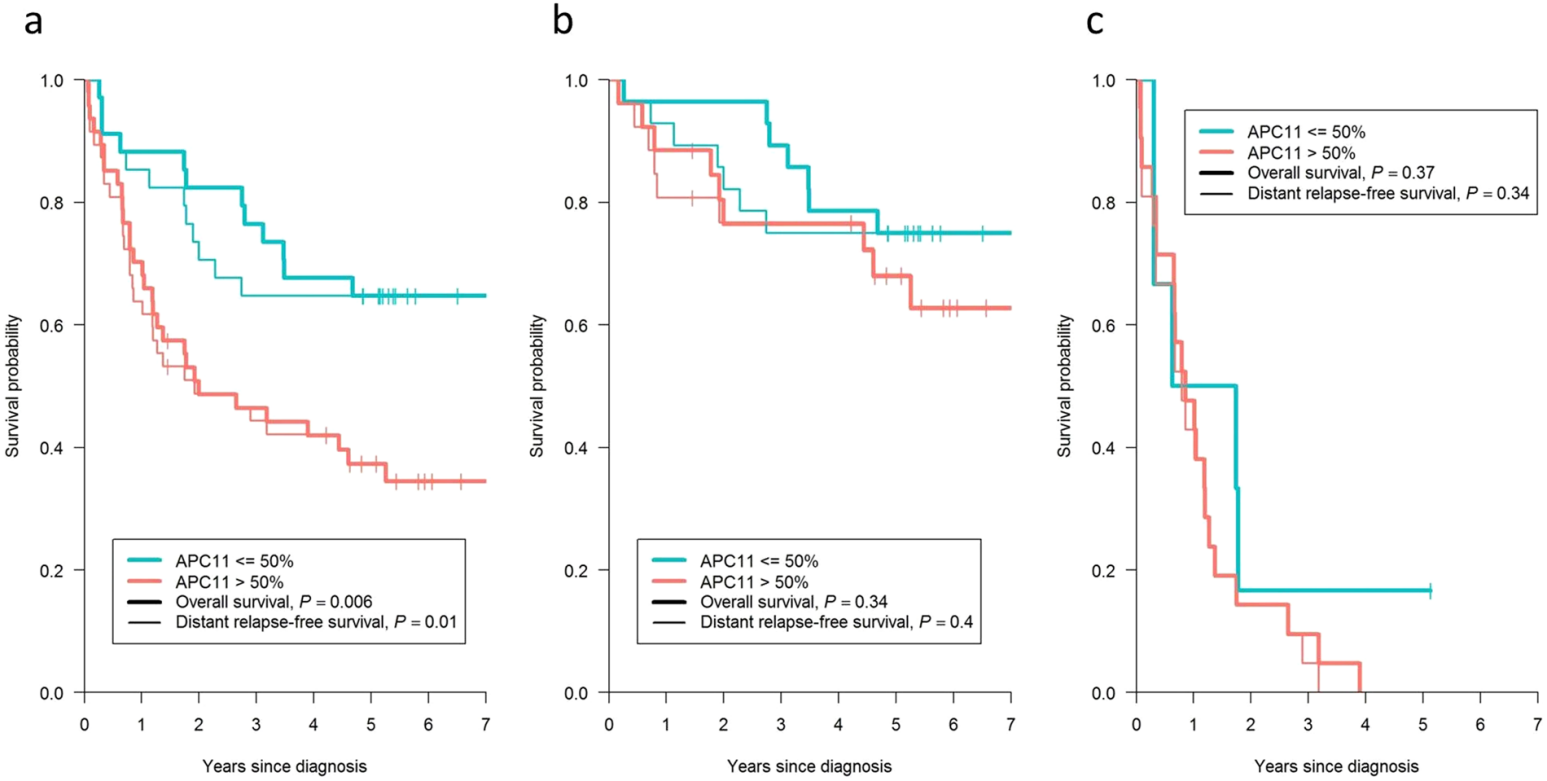
Kaplan-Meier curves presenting the probability of CRC patient survival according to APC11 protein expression. (**a**) All patients (n = 81). (**b**) Patients with M0 disease (n = 54). (**c**) Patients with M1 disease (n = 27). *P* values displayed were calculated by the log-rank test. M1: patients with metastasis at CRC diagnosis; M0: patients without metastasis at CRC diagnosis.

Unadjusted and adjusted logistic regression models (Table 3) identified likely statistical associations between elevated APC11 protein expression (>50% marked cells) and the presence of tumor residue after surgery (adjusted Odds ratio, OR = 6.51; 95% CI = 1.54–27.59; *P* = 0.012), metastasis at diagnosis (adjusted OR = 3.87; 95% CI = 1.20–12.45; *P* = 0.024), and indicated possible associations with vascular invasion (adjusted OR = 2.96; 95% CI = 0.88–9.96; *P* = 0.079), node involvement (adjusted OR = 2.48; 95% CI = 0.89–6.94; *P* = 0.082), TNM stage (adjusted OR = 2.33; 95% CI = 0.84–6.43; *P* = 0.10), pre-operative CEA (adjusted OR = 2.66; 95% CI = 0.72–9.75; *P* = 0.14) and tumor size (adjusted OR = 2.29; 95% CI = 0.60–8.84; *P* = 0.22). Of note, the adjusted ORs were slightly lower but remained close to unadjusted ORs, suggesting the relative independence of APC11 compared to the combined effect of the other protein markers. Survival analyses showed that patients with a high level of APC11 protein expression also had a worse overall survival (OS) (adjusted Hazard ratio, HR = 2.69; 95% CI = 1.31–5.51; *P* = 0.007) and a worse distant relapse-free survival (DRFS) (adjusted HR = 2.60; 95% CI = 1.26–5.37; *P* = 0.01) (Table 3 and Fig. 5a). However, Cox models stratified according to the presence of metastasis at diagnosis (Table 3) and Kaplan Meier graphs separating patients with M0 disease (Fig. 5b) from patients with M1 disease (Fig. 5c) showed that these poorer survival rates in patients with elevated APC11 levels probably came from their metastatic statuses at diagnosis rather than metastatic relapses.

**Table 3:** Association of APC11 >50% with clinical and survival outcomes.

Clinical outcomes	N^a^	Effect of APC11 >50% marked cells			*P*
		Unadjusted OR (95% CI)	*P*	Adjusted^b^ OR (95% CI)	
Tumor residue: R1-R2 vs. R0	82	6.77 (1.81–25.31)	0.0045	6.51 (1.54–27.59)	0.012
Metastasis pM: M1 vs. M0	82	3.95 (1.38–11.27)	0.010	3.87 (1.20–12.45)	0.024
Vascular invasion: presence vs. absence	80	3.92 (1.36–11.26)	0.011	2.96 (0.88–9.96)	0.079
Tumor size pT: T3-T4 vs. T1-T2	81	3.35 (1.10–10.23)	0.034	2.29 (0.60–8.84)	0.22
Pre-operative CEA: increased vs. normal	66	3.01 (1.04–8.73)	0.043	2.66 (0.72–9.75)	0.14
Stage TNM: III-IV vs. I-II	82	2.47 (1.00–6.07)	0.050	2.33 (0.84–6.43)	0.10
Node involvement pN: N1-N2 vs. N0	81	2.47 (1.00–6.15)	0.051	2.48 (0.89–6.94)	0.082
Differenciation: poor vs. good-moderate	82	1.95 (0.73–5.22)	0.18	1.62 (0.54–4.85)	0.39
Ploidy: aneuploid vs. diploid	70	1.13 (0.41–3.11)	0.81	0.99 (0.30–3.27)	0.98
Tumor location: right-transv. vs. left-up-rectum	81	0.83 (0.33–2.09)	0.70	0.71 (0.24–2.09)	0.53
Stroma: not lymphoid vs. lymphoid	68	0.43 (0.16–1.17)	0.098	0.38 (0.12–1.23)	0.10
**Survival outcomes**	**N** ^**a**^	**Unadjusted HR (95% CI)**	***P***	**Adjusted** ^**b**^ **HR (95% CI)**	***P***
Overall survival	81	2.49 (1.27–4.87)	0.008	2.69 (1.31–5.51)	0.007
*Cox model stratified by M status*	81	1.59 (0.79–3.20)	0.2	1.83 (0.84–4.02)	0.13
Distant relapse-free survival	81	2.36 (1.20–4.61)	0.012	2.60 (1.26–5.37)	0.01
*Cox model stratified by M status*	81	1.56 (0.78–3.14)	0.21	1.85 (0.84–4.07)	0.12

^a^Number of patients with available data. ^b^Adjusted ORs and HRs were obtained from multivariable logistic and Cox regression models including APC11, E-cadherin, KI67, MLH1, MSH2, DCC, P53 and BCL2. Abbreviations: OR, odds-ratio; CI, confidence interval; HR, Hazard ratio.

**Figure 6:**
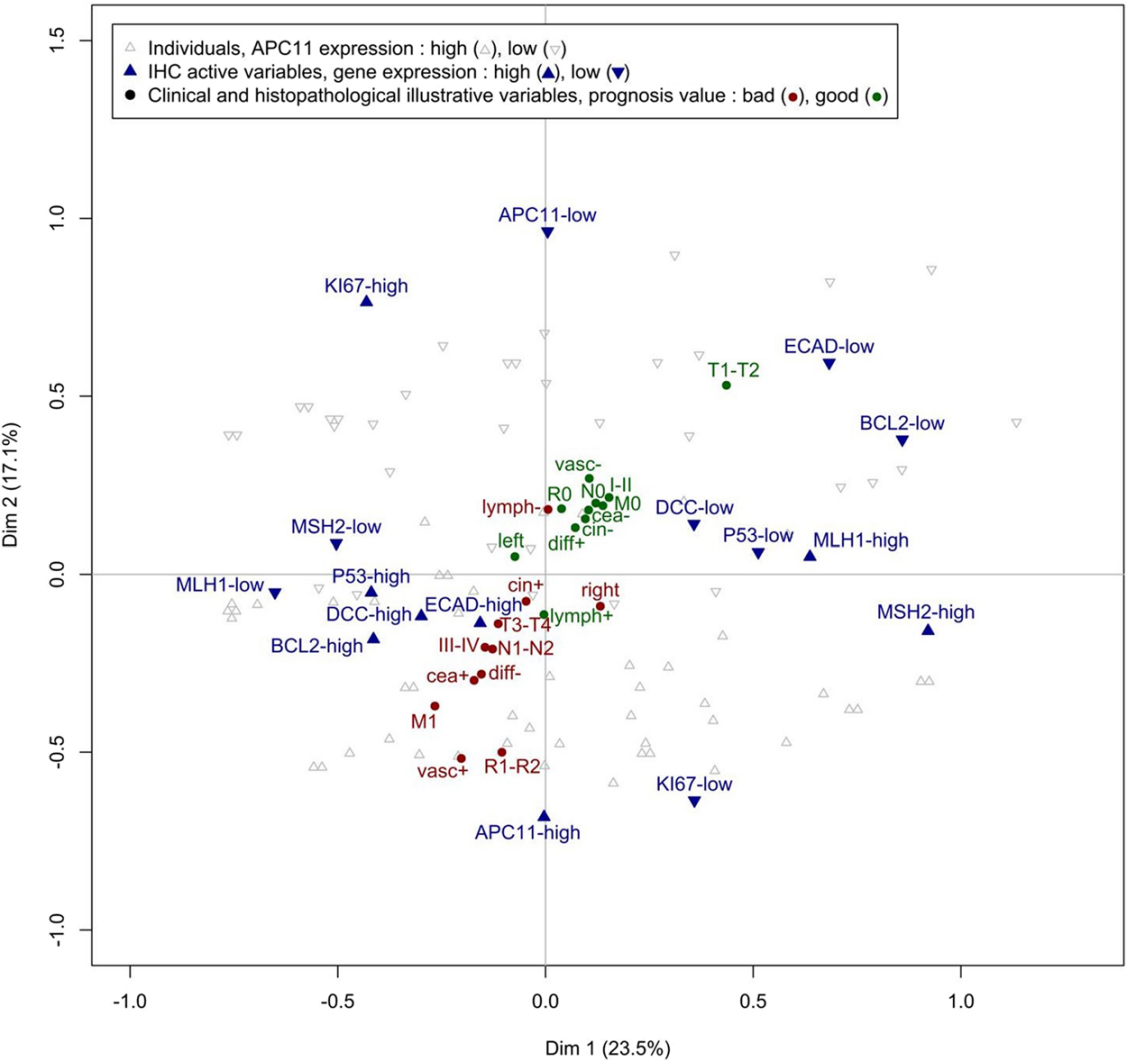
Multiple Correspondence Analysis (MCA) of immunohistochemical and clinical data. The MCA suggests that protein expression of APC11 is independent of other protein markers (due to their orthogonal projection on the figure). As clinical data is projected on the diagonal, the MCA also suggests that APC11 is correlated with the clinical data independently of the other protein markers. Labels used: tumor residue: R0/R1-R2; metastasis pM: M0/M1; vascular invasion: vasc+: presence/vasc−: absence; pre-operative CEA: cea+: increased/cea−: normal; node involvement pN: N0/N1-N2; stage TNM: I-II/III-IV; tumor size pT: T1-T2/T3-T4; differentiation: diff−: poor/diff+: good-moderate; stroma: lymph+: lymphoid/lymph−: not lymphoid; ploidy: cin+: diploid/cin−: aneuploid; tumor location: right: right-transverse/left: left-up-rectum.

In order to supplement the above results obtained from logistic regression models, we also performed a Multiple Correspondence Analysis (MCA), which offered a remarkable picture highlighting the correlations between protein markers and clinicopathological data (Fig. 6). The 8 protein markers could be organized into two groups: the first being constituted of E-cadherin, Bcl2, MLH1, MSH2, DCC and P53 (principal contributors of Dim 1), and the second of APC11 and Ki67 (principal contributors of Dim 2). Interestingly, when projecting clinical and histopathological data, a diagonal of the disease’s severity emerged, ranging from good prognosis criteria projected in the top right-hand corner (e.g., T1-T2) to bad prognosis criteria in the bottom left-hand corner (e.g., M1), illustrating that APC11 is correlated with clinical outcome independently of the other protein markers as previously suggested by multivariable regression models.

#### Association between protein levels and simplified CMS group classification

We designed a simplified classification based on the molecular and clinical characterization of the CMS groups recently published by Guinney *et al*.^12^ (see Supplemental Methods for details). The resulting study-specific CMS classification is depicted in Table S3 and Fig. S7. OS and DRFS were worse for the study-specific CMS groups 1 and 4 (Fig. S8). The APC11 protein expression level was not associated with the study-specific CMS classification and thus appeared as a likely independent marker (Fisher’s exact test: *P* = 0.39, Table S3).

## Discussion

In the present work we uncovered a novel and likely predictive marker of colorectal cancer, namely APC11, the key enzymatic subunit of the anaphase promoting complex, using an innovative integrated analysis incorporating relevant protein markers and clinical variables. Through multivariable regression models and multiple correspondence analysis (MCA), we evaluated the potential role of APC11 as a prognostic marker independent of 7 key CRC biomarkers. These included (i) MLH1 and MSH2, since they represent major markers for identifying MSI+ colorectal tumors. Indeed, it was previously shown that lower MLH1 and MSH2 protein levels were associated with reduced recurrence rates and better survival^27^. (ii) Deleted in colorectal cancer (DCC), the expression of which is highly associated with the CIN genotype in CRC. As DCC is also subjected to epigenetic silencing^31^ and not only to loss of heterozygosity (LOH), it appears relevant to analyze its deregulation in CRC at the protein level. In colorectal tumorigenesis, loss of DCC expression follows the activation of the KRAS oncogene and is associated with a transition from intermediate adenoma to late adenoma^32^. *DCC* gene location corresponds to one of the most frequent genetic abnormalities that occur in advanced CRC, namely LOH in the 18q21 region. However, the 18q allelic imbalance (AI) may be a non-specific surrogate marker for CIN making it difficult to take into account a lower expression pattern of DCC as a predictive biomarker. Different studies have demonstrated that CIN was associated with a worse prognosis in CRC^33^. (iii) P53 could be a relevant biomarker in CRC, since mutations in *TP53* gene resulting in protein overexpression, are frequently observed in CRC and often associated with CIN. (iv) Cancer cell proliferation was assessed using the nuclear proliferation marker Ki67 protein expression. (v) Bcl2 protein expression, because it was surprisingly shown that low Bcl2 levels were correlated with an increase in the number of relapses in stage II CRC, and high levels of Bcl2 protein seem to be associated with slower local tumor growth^34^. (vii) Lastly, E-cadherin down-regulation was shown to be strongly associated with the invasive potential of the tumor and mCRC^35^.

From our analyses, APC11 protein expression appeared to be independent of the other known protein markers studied, and thereby might be a new independent predictive marker. Indeed, we found that patients exhibiting high levels of APC11 protein had poor clinical outcomes and survival rates, even after statistical adjustment for the effects of the other markers.

Guinney *et al*.^12^ proposed to categorize CRC into 4 consensus molecular subtypes (CMS); the CMS1 (14%) called MSI-like corresponds to CRC tumors with mutations in mismatch repair (MMR) genes, such as MLH1 and is also enriched in the CpG island methylation phenotype (CIMP). CMS1 displays mutations in the *BRAF* gene, shows strong immune activation and infiltration, and worse survival after relapse. The CMS2 subtype, called canonical (37%), contains tumors of epithelial phenotype exhibiting CIN, with WNT and Myc activation. The CMS3 subtype, called metabolic (13%), corresponds to tumors with an epithelial phenotype and dysregulations in their metabolic pathways, and is enriched in *KRAS* mutations and a mixed MSI+/CIN genetic status. Finally, the CMS4 subtype, called mesenchymal (23%), frequently harbors a CIN genetic status, displays prominent TGF *β* pathway activation, stromal infiltration, angiogenesis and worse OS and relapse free-survival (RFS). Here, we designed a study-specific CMS classification, compiling and selecting 12 discriminating binary key elements of the CMS classification (Table S2). Our results suggest that the expression of the APC11 protein in primary CRC might be independent of the CMS subtypes (Table S3). Moreover, our study-specific CMS subtyping for OS and DRFS match the OS and RFS presented by Guinney *et al*.^12^ for CMS 2, 3 and 4, but not for CMS1. As in our study DRFS encompasses both RFS and SAR (survival after relapse), which are considered separately in Guinney *et al*., this may explain the divergence between the two studies. This discrepancy might also be due to misclassifications in our study-specific CMS subtyping. Notably, our study-specific CMS classification resulted in 21% of “Indeterminate” tumor samples compared to only 7% in the work by Guinney *et al*. (See Supplemental data). Evaluating the interest of APC11 with regards to the current CMS classification requires further studies and at this point, APC11 expression can’t be considered as a key element to refine or modify molecular CRC classification.

We also characterized the levels of APC11 regarding the chromosomal alterations status of CRC, including analyses of experimental data and public CCLE^29^ and TCGA^30^ datasets, and identified a strong correlation between high levels of APC11 and chromosomal instability. In the CMS classification, enriched cell cycle and proteasome transcriptomic signatures were found both in the CMS2 canonical subtype (CIN) and in the CMS1 MSI immune subtype^12^. Over-enrichment of mutations in cancer drivers is seen in CMS1, with the exception of the 2 tumor suppressor genes *APC* and *TP53*. *APC* mutations are significantly enriched in CMS2, while *TP53* mutations are enriched both in CMS2 and CMS4 subtypes, highly associated with CIN status. Loss of the tumor suppressor *APC* leads to CIN by disrupting the function of the MCC and limiting kinetochore-microtubule interactions. The involvement of p53 in sensing mitotic failure has been largely described in particular in the regulation of MAD1 expression. Nevertheless, the relationship between p53 and APC/C was much less scrutinized. Interestingly, it was shown that p53/p21-genotoxic stress induction at G2 phase triggers APC/C-Cdh1 activation^17^. APC/C-Cdh1 activation upon replication stress is also p53/p21-dependent^36^, and has been involved in DNA damage response by shutting down DNA repair machinery after DNA repair completion^17^. In turn, as a feedback mechanism, it was reported that APC/C-Cdc20 activates the degradation of p21^37^. These results suggest that p53 inactivation may abrogate the APC/C-Cdh1 activation response and may render cancer cells more permissive to Cdc20 overexpression. A recent study has shown that 39% (vs 28%) of tumors with an APC/C mutation also harbors a *TP53* mutation in a cohort of nine cancer types analysed^28^. In CRC cell lines, high expression of APC11 appeared to be associated with mutated *TP53* in our first analyses by RT-qPCR (*P* = 0.059), however this result could not be replicated in public datasets.

Alongside the presence of mutations, variation in some APC/C core subunits expression may disrupt the stochiometry of the complex thus affecting chromosome stability during tumor progression. Very few studies have reported alteration in APC/C core subunits expression in cancer. Shi and Huo^38^ have performed *APC11* siRNA in HEK293T cells and shown that reduced APC11 levels by over 50% led to a decrease number of cells in G2/M phase and a higher number in G1 phase, while overexpression of APC11 led to an increase in the number of cells in G2/M. Complete inhibition of APC/C core subunits expression has been shown to be detrimental for cell survival as it was reported that KO models of the three catalytic subunits APC2, APC10 and APC11 were embryonic lethal^39–41^. Of note, complete ablation of APC/C activity causes cohesion fatigue in cells harboring a functional SAC, which results in a mitotic cell death. Rather a complete inhibition of APC/C activity, a reduction in this ubiquitin ligase E3 activity in cancer cells together with SAC impairment may induce mild delays in mitosis. p53 inactivation and dysregulated APC/C may confer a selective advantage in SAC impaired tumors by reducing the rate of segregation errors to reach an equilibrium and to keep genomic instability at a sustainable ratio^28^. Thus, APC/C alterations may occur late in tumor progression following CIN onset. Down or up-regulation of APC/C core subunits expression may have the same impact in cancer cells as it was reported that both CDC27 overexpression and CDC27 haploinsufficiency in CRC are correlated with poor patient survival^42^. CDC27 haploinsufficiency is one of the 23 cancer driver genes identified in CRC^30,43^. *APC2* mRNA expression was also investigated in different types of cancer and decreased levels were associated with cancer progression^44^. It was shown that APC2 deficiency results in mdm2 protein increased levels and subsequent p53 inactivation^44^. Very little is known about APC/C subunits transcriptional regulation; interestingly Cdc20 was shown to be transcriptionally down-regulated by the tumor suppressor protein p53 upon DNA damage^45^. At post-transcriptional level, SNW1 splicing factor regulates the splicing of both *APC2* and *APC11* pre-mRNA^46^. More studies have focused on post-translational regulation of APC/C subunits and have demonstrated that APC/C is regulated by various post-translational modifications notably phosphorylation, sumoylation, and acetylation^47–50^. Interestingly, it was shown that the Polycomb Repressive Complex 1 (PRC1) subunit PSC, involved in self-renewal of cancer stem cells, interacts specifically with APC11 to ubiquitylate cyclin B cooperatively^51^.

So far, no previous work has reported altered APC11 expression in cancer. Incorporating APC11 protein expression into a clinical context, may allow medical oncologists to refine the selection of CRC patients who might benefit from taxane chemotherapy, which previously failed to show therapeutic advantages when administered randomly, irrespective of molecular signatures. Dysregulation of the APC/C destruction pathway in SAC efficient tumors might represent a potentially important cancer-specific therapeutic vulnerability. Indeed, APC11 overexpression may lead to mitotic cell death of taxane-treated cancer cells, suggesting that high levels of APC11 may increase drug cytotoxicity. Giovinazzi *et al*.^52^ reported that proTAME APC/C inhibitor (inhibition of binding between core APC/C, Cdc20 and substrate) prohibited mitotic exit of paclitaxel treated cells. Sackton *et al*.^53^ revealed combined use of Apcin (disruption of the interaction between Cdc20 and substrate) and proTAME to increase duration of mitosis and block its exit. APC/C inhibitors therapies should be carefully considered since the APC/C inhibitor proTAME can rescue segregation errors in SAC impaired cells^28,52^. Conversely, functional APC/C in SAC impaired tumors may increase CIN to a lethal level. Clinical management of CRC is still based on TNM classification for therapeutic decisions, and APC11 protein expression may thus provide a novel, cost-effective, immunohistochemistry-based means of improving personalized therapeutic strategies.

## Methods

### Cell lines

Twenty-one different colon cancer cell lines were included in this study (Table S4). Twelve were obtained from the American Type Culture Collection (http://www.atcc.org), EB was kindly provided by Philip Shaw (IUP, Lausanne, Switzerland), and Co-115, Isreco1 (IS1), Isreco2 (IS2), Isreco3 (IS3), TC-7 and TC-71 by Richard Hamelin (INSERM, UMRS 938-Centre de Recherche Saint-Antoine, Paris, France). The cell lines IS1, IS2 and IS3 were derived from a primary colon carcinoma and from the corresponding liver and peritoneal metastases in the same patient, respectively^54,55^. Other cell lines were derived from human primary colon carcinomas. The HME-1 (normal human mammary epithelial) cell line was used as a control for RT-qPCR analyses (Lonza, Basel, Switzerland). Colorectal cell lines were cultured in Dulbecco’s Modified Eagle’s Medium (DMEM) with added fetal bovine serum, glutamine, penicillin and streptomycin, and were maintained in humidified 37 °C 5% CO2 incubators. Cells were grown to 90% confluence according to the ATCC protocols (http://www.atcc.org).

#### Total RNA isolation and cDNA synthesis

Total RNA was isolated from cells using TRI Reagent (Sigma Chemical Co., Saint Louis, MO, USA). This RNA, which was largely free of contaminating DNA, was further purified using Phase-Lock gel tubes (Eppendorf, Le Pecq, France). Total RNA concentration was determined spectrophotometrically at 260 nm (Spectrophotometer UV-VIS, DU-700, Beckman Coulter Inc., Brea, California, USA), while RNA integrity was tested on a 1% agarose gel. cDNA was synthesized from 1 *μ*g of total RNA using a first-strand cDNA synthesis kit (Amersham Pharmacia Biotech, Uppsala, Sweden), according to the manufacturer’s instructions. Total RNAs from 2 normal adult colon tissues were obtained from the BioChain Institute (Newark, USA) and served as controls.

#### Real-time quantitative PCR (RT-qPCR) analysis

*APC11* mRNA levels were assessed by conducting a two-step RT-qPCR in a LightCycler (Roche Molecular Biochemicals, Applied Science, Basel, Switzerland). Specific primer pairs used for PCR amplification were available commercially (Roche Applied Biosystems). All reactions were performed in glass capillaries (Roche Molecular Biochemicals) using the LightCycler FastStart DNA Master PLUS SYBR Green I kit and deionized water as a negative control. The Thermocycling program was as follows: 95 °C for 10 min, (45 cycles) at 95 °C for 10 s, 55 °C for 10 s, 72 °C for 6 s, 45 °C to 95 °C by increments of 0.1 °C, followed by a cycle at 40 °C for 30 s. All standards and samples were analyzed in duplicate and experiments were repeated at least three times. Fluorescence data were analyzed with the LightCycler 4.0 software (Roche Applied Science, Basel, Switzerland). cDNA prepared from the HME-1 cell line served as a calibrator for all qPCR reactions. For relative quantification and normalization, the comparative Ct (or E- where E is the primer-dependent efficiency of the PCR) method was used. The Ct values of both the calibrator and the samples of interest were normalized using 3 housekeeping genes, *PPIB*, *β-Actin* and *PGK*. The primer pairs and probes were designed using the Universal Probe Library website (Roche Applied Science). Primers of each pair were located in different exons to avoid genomic amplification. Primer pairs are listed in Supplemental Table S5. For relative quantification and normalization, the following calculation was used:

1 $$N={E}_{T}^{CpT(C)-CpT(S)}\times \prod ({E}_{R}^{CpR(S)-CpR(C)}),$$

in which *N* is the normalized ratio, *E* the efficiency, *Cp* the crossing point, *T* the target gene (*APC11* with *E* = 1.763), *R* the reference gene (*PPIB* with *E* = 2.000, *ACTB* with *E* = 1.920, *PGK-1* with *E* = 1.994), *C* the calibrator (HME-1) and *S* the unknown sample^56^. PCR efficiencies were calculated in triplicate using relative standard curves derived from serial dilutions (from 1/6 to 1/60,000) of the calibrator.

#### Western blot analysis

Cell pellets were lyzed in RIPA buffer for 30 min on ice and then centrifuged 30 min at 13,000 rpm at 4 °C. The protein concentration of the supernatant was determined by performing a Bradford assay using the Biorad Protein assay (Biorad, Marnes-la-Coquette, France) and BSA as a standard. Western blot analysis was performed as previously described^57^. APC11 antibodies (monoclonal antibodies (M01), clone 1B4-1A4 reference H00051529-M01, Abnova, Taipei, Taiwan) were diluted at 1/500 and *β*-actin antibodies (clone 14, Becton Dickinson) were diluted at 1/2,000. After 1 h incubation, membranes were washed 3 times with TBS 1X-Tween 0.5%. The secondary antibodies (P0217, Dako, Trappes, France) were diluted at 1/3,000 in the same buffer and incubated for 1 h. APC11 was detected at 9.8 kDa using the Lumi-LightPLUS (Roche, Applied Science, Basel, Switzerland).

### Clinical samples and clinical data

One hundred and ninety one colorectal samples were collected and arranged in tissue microarrays (TMAs), kindly provided by Dr Geneviéve Monges at the Department of Anatomic Pathology of the Paoli-Calmettes Cancer Centre (Comprehensive Cancer Center of Marseille, France). The samples were collected before therapy from 99 male and 92 female patients diagnosed with colorectal cancer between 1990 and 1998, who were undergoing surgery at the Paoli-Calmettes Centre. All of the patients gave their informed consent before the removal of biological tissues for the purpose of research studies. Furthermore, the collection of human tissue samples received approval by the ethics committee“Comité de Protection des Personnes” (Paoli-Calmettes Cancer Centre of Marseille)^26^. Patient cohorts, tumor characteristics and immunohistochemical covariates (E-cadherin, Ki67, MLH1, MSH2, DCC, p53 and Bcl2) were previously published^26,27,34,58,59^. Information on the evolution of the tumor in terms of local or distant recurrences was registered prospectively. Overall, complete IHC comparative data could only be obtained for 82 individuals. Normal colon tissue samples were obtained from the Institutional Biological Resources Department of the Centre Léon Bérard (Comprehensive Cancer Center of Lyon, France) (agreement number DC-2008-99). All experiments were performed in accordance with relevant guidelines and regulations.

#### Immunohistochemistry (IHC)

Core specimens were taken in triplicate from formalin-fixed paraffin-embedded colon tumors and were arranged into TMA blocks as previously described^60^. Normal colon tissue samples from the Centre Léon Bérard were analyzed on full tissue sections. 5 *μ*m TMA tissue sections were deparaffinized and rehydrated. The slides were then incubated in 5% hydrogen peroxide in sterile water to block the activity of endogenous peroxidases. No antigen retrieval was performed for rabbit polyclonal anti-APC11 antibody (Ab133200, Abcam, Cambridge, UK). Slides were incubated for 1 h at room temperature with the primary antibody diluted 1/50 in an antibody diluent solution (Chem Mate, Dako, Trappes, France). After rinsing in PBS, the slides were incubated with a biotinylated secondary antibody bound to a streptavidin peroxidase conjugate (Vectastain Elite ABC reagent Vector, Abcys, Paris, France). Bound antibody was detected by adding the substrate 3,3′-diaminobenzidine (LSAB+ substrate kit for peroxidase, K675 Agilent Pathology Solutions). The sections were counterstained with hematoxylin. A pathologist and a technician analyzed IHC stained tissues independently. Both the intensity of cytoplasmic staining (3 grades) and the percentage of positive cells were assessed. The intensity of staining was graded on a 3 point scale from 0 to 2: “0” reflected lack of immunoreactivity, “1” weak immunoreactivity and “2” strong immunoreactivity. Once the two investigators reached a consensus on some samples, they reviewed cases with discordant scores.

### Public data from CCLE and TCGA repository

Segmented copy-number data, mutation and mRNA expression data were obtained from the CCLE website^29^ for the 59 CRC cell lines with mRNA data available, and from the cBio Cancer Genomics Portal^61^ for the 174 rectal (READ) and 499 colon (COAD) tumour samples of TCGA repository. Corresponding clinical data were extracted using the CGDS-R package provided by the cBio Cancer Genomics Portal to query the Cancer Genomics Data Server^30^.

### Statistical analyses

#### mRNA expression in cell lines experiments

The log_2_ mean mRNA expression of CRC cell lines was estimated using the following random effects model:

2 $${\mathrm{log}}_{2}({Y}_{ij})=\mu +{\alpha }_{i}+{\varepsilon }_{ij},$$

in which *Y*_*ij*_ is the normalized APC11 RNA expression for measurement *j* of cell line *i*, *μ* is the overall mean across all samples and replicates after log_2_ transformation, *α*_*i*_ represents the random effects associated with cell lines and ${\varepsilon }_{ij}$ the unexplained error. The statistical significance of the *α*_*i*_ term (i.e. biological variation) was estimated using a forward likelihood profile analysis based on the likelihood ratio (LR) test. Estimations were conducted using the maximum likelihood method and the nlme library of the R software^62^. For likelihood ratio tests, the statistical significance level was set at *P* < 0.05.

#### Analyses of IHC and clinical/histopathological data

The statistical association between the level of APC11 measured by IHC and the clinical/histopathological data was assessed by logistic regression models, evaluating various clinical variables one after the other as the outcome variable (i.e., dependent variable), providing unadjusted odds-ratios (ORs) for the effect of APC11. Adjusted odds-ratios (ORs) were obtained by including 7 key biomarkers, namely E-cadherin, Ki67, MLH1, MSH2, DCC, p53 and Bcl2 as explanatory variables (i.e., independent variables) in the regression models. The positive threshold for APC11 protein expression was arbitrarily set at >50% marked cells, prior to any statistical analysis. Missing data were handled using list wise deletion for clinical/histopathological data (outcome variables) and using a multiple imputation approach for the 7 biomarkers used as adjustment variables so that unadjusted and adjusted estimates were produced on the same series of patients^63,64^ (see details in the Supplemental material). Kaplan-Meier curves and the log-rank test were performed to analyze OS and DRFS stratified according to the expression of APC11 and the metastatic status of patients at diagnosis. OS was defined as the time elapsed from initial diagnosis to the last observation or to death from CRC. DRFS was defined as the time elapsed from initial diagnosis to metastatic relapse or death from CRC, or last observation if the patient was alive without metastasis. Cox regression models were fitted to estimate the effect of APC11 on OS and DRFS by unadjusted and adjusted hazard ratios (HRs). A Multiple Correspondence Analysis (MCA) was performed to describe graphically and synthetically the correlations between protein data measured by IHC. Protein data were used as active variables whereas clinical and histopathological data were used as illustrative variables (i.e. not contributing to the construction of the dimensions) to offer a global and integrated picture of the protein and clinical/histopathological data.

#### Analyses in the CCLE and TCGA public datasets

The FGA (fraction genome altered) was calculated from segmented copy-number data as the ratio of the sum of the lengths of all segments with signal above a predetermined threshold to the sum of all segment lengths. Due to contamination with non-tumour material in TCGA tumour samples, a threshold value of 0.2 was used for TCGA tumour samples and a threshold value of 0.3 was used for the CCLE cell lines which are purer^65^. Correlation between mRNA expression and the FGA was quantified with Pearson’s correlation coefficient and with both Wilcoxon’s test and Student’s t-test comparing expression levels of the samples at the two extremes quartiles of the FGA (Q75 vs Q25). Sensitivity analyses were performed varying the threshold used for the FGA calculation from 0.1 to 0.6. Further analyses were carried out in the TCGA data. Kruskal-Wallis and Anova F-test were used to investigate statistical association between mRNA expression levels and tumour staging, lymphovascular invasion and residual tumor statuses. Kaplan-Meier curves and the log-rank test were performed to analyse Overall Survival (OS) stratified according to the expression of *APC11* and the metastatic status of patients at diagnosis.

### Data Availability Statement

The datasets generated during and/or analysed during the current study are available from the corresponding author on reasonable request.

## Electronic supplementary material

Supplemental material
