# Supplementary material for: Integrated analysis highlights APC11 protein expression as a likely new independent predictive marker for colorectal cancer
Source: Sci Rep. 2018 May 9;8:7386. doi: 10.1038/s41598-018-25631-1 (PMC5943309; doi:10.1038/s41598-018-25631-1)
Supplement: Supplementary file 1 — Supplemental material [file 41598_2018_25631_MOESM1_ESM.pdf]

Integrated analysis highlights APC11 protein expression as a likely new independent predictive marker for colorectal cancer.

Youenn Drouet, Isabelle Treilleux, Alain Viari, Sophie Léon, Mojgan Devouassoux-Shisheboran, Nicolas Voirin, Christelle de la Fouchardière, Brigitte Manship, Alain Puisieux, Christine Lasset, Caroline Moyret-Lalle.

Supplemental Methods

Treatment of missing values for IHC data

Among the 82 patients with available APC11 expression by IHC, 64 (78%) patients had no missing value for the 7 other biomarkers measured by IHC, 15 (18%) patients had a missing value for one biomarker, and 3 patients (4%) had missing values for two biomarkers. In order to perform univariable and multivariable analyses to the same series of patients, these missing values were imputed using the iterative regularized MCA algorithm[1], by assuming that missing values were "missing at random" (MAR). To propagate the uncertainty linked to this imputation step to the final estimates and their confidence intervals and p-values, we devised a multiple imputation approach following the theory developed by Rubin[2]. This consisted in generating 1,000 complete datasets using the missMDA R library[3], analyzing these datasets independently and pooling the results using the mice R library[4]. This imputation step was also performed prior to conducting the MCA analysis in order to being able to project all the individuals on the factorial axes.

Study-specific classification for the CMS groups

We devised a study-specific classification for the CMS groups using the molecular and clinical characterization of the CMS groups recently published by Guinney *et al.*[5]. Each CMS group was first characterized by 12 discriminating binary variables measured in our study, using the values 1 and 0 for respectively the most and least likely categories or the value 0.5 when both categories were assumed equally likely (Table S2). For example, we defined the CMS1 group as most likely MLH1- and MSH2- because of the high proportion of high microsatellite instability (MSI) values for this group reported by Guinney *et al.* [5, see fig. 3c]. From this study-specific characterization of the CMS groups we then computed for each individual of our study four statistical distances (one for each CMS group) defined as the following euclidean distance:

$$D_{ij} = \sqrt{\sum_{k=1}^{22} (x_{ki} - y_{kj})^2}, \tag{1}$$

where x_{ki} and y_{kj} correspond respectively to the values of the category k measured in patient i and defined for the CMS group j. A similarity measure was then defined as:

$$S_{ij} = 1/(1 + D_{ij}). \tag{2}$$

A study-specific classification for the CMS groups was then obtained by attributing to each patient the most proximate CMS group, that is, the one that maximized the similarity value S_{ij} :

$$CMS_i = \arg\max_j (S_{ij}). \qquad (3)$$

Classification was defined as "indeterminate" when more than one CMS group got the maximum value for S_{ij}. The resulting study-specific classification is depicted in Table S3 and Figure S3. Overall survival and distant relapse-free survival were worse for the CMS1 and CMS4 groups (Figure S3).

References

1. Josse, J., Chavent, M., Liquet, B. & Husson, F. Handling Missing Values with Regularized Iterative Multiple Correspondence Analysis. *J. Classif.* **29**, 91–116 (2012).
2. Rubin, D. B. (ed.) *Multiple Imputation for Nonresponse in Surveys*. Wiley Series in Probability and Statistics (John Wiley & Sons, Inc., Hoboken, NJ, USA, 1987).
3. Josse, J. & Husson, F. missmda: A package for handling missing values in multivariate data analysis. *J. Stat. Software, Articles* **70**, 1–31 (2016).
4. van Buuren, S. & Groothuis-Oudshoorn, K. mice: Multivariate imputation by chained equations in r. *J. Stat. Software, Articles* **45**, 1–67 (2011).
5. Guinney, J. *et al.* The consensus molecular subtypes of colorectal cancer. *Nat. Medicine* **21**, 1350–1356 (2015).

Supplemental Tables and Figures

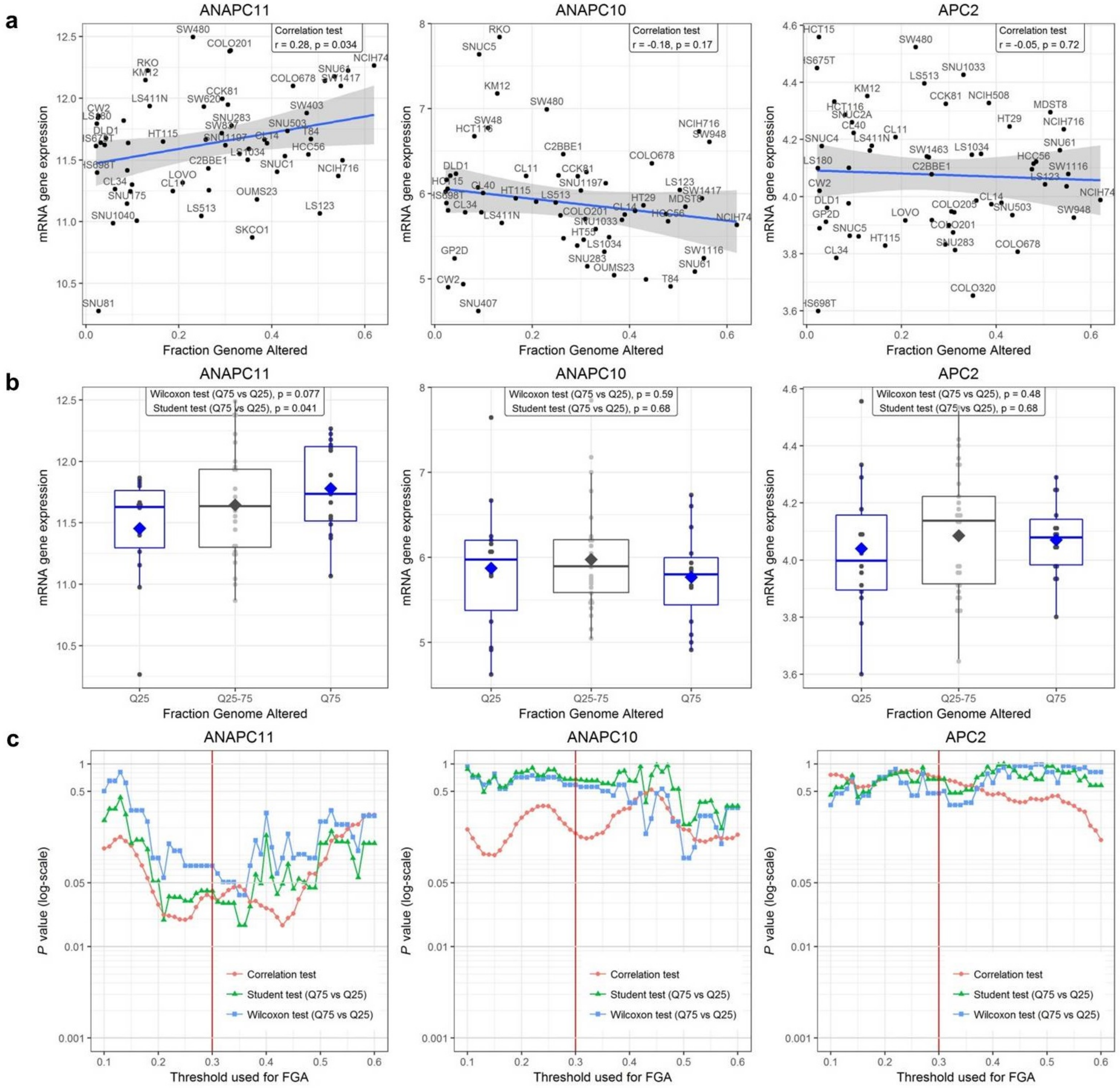

Figure S1. Analysis of the mRNA gene expression of *APC11*, *APC10* and *APC2* in 59 CRC cell lines from the CCLE dataset. (a) For each gene, the top panel shows the gene expression according to the fraction of genome altered (FGA) calculated with a threshold value of 0.3. The coefficient of correlation r is displayed with the corresponding *P* value; The regression line from a linear model (blue line) and its 95% confidence interval (grey area) are also displayed. **(b)** The middle panel shows the comparison of the gene expression levels between the two extremes quartiles Q75 and Q25 of the FGA, with the non-parametric Wilcoxon test and the Student t-test. The means of the expression levels at the two extremes quartiles are indicated by blue diamonds. **(c)** The bottom panel displays the results of a sensitivity analysis for the threshold used for the FGA calculation. The p-values of the three tests (correlation, Wilcoxon and Student) are shown as a function of the threshold used for the FGA calculation. The vertical red lines indicate the value of the threshold that were used for the FGA calculations in a) and b).

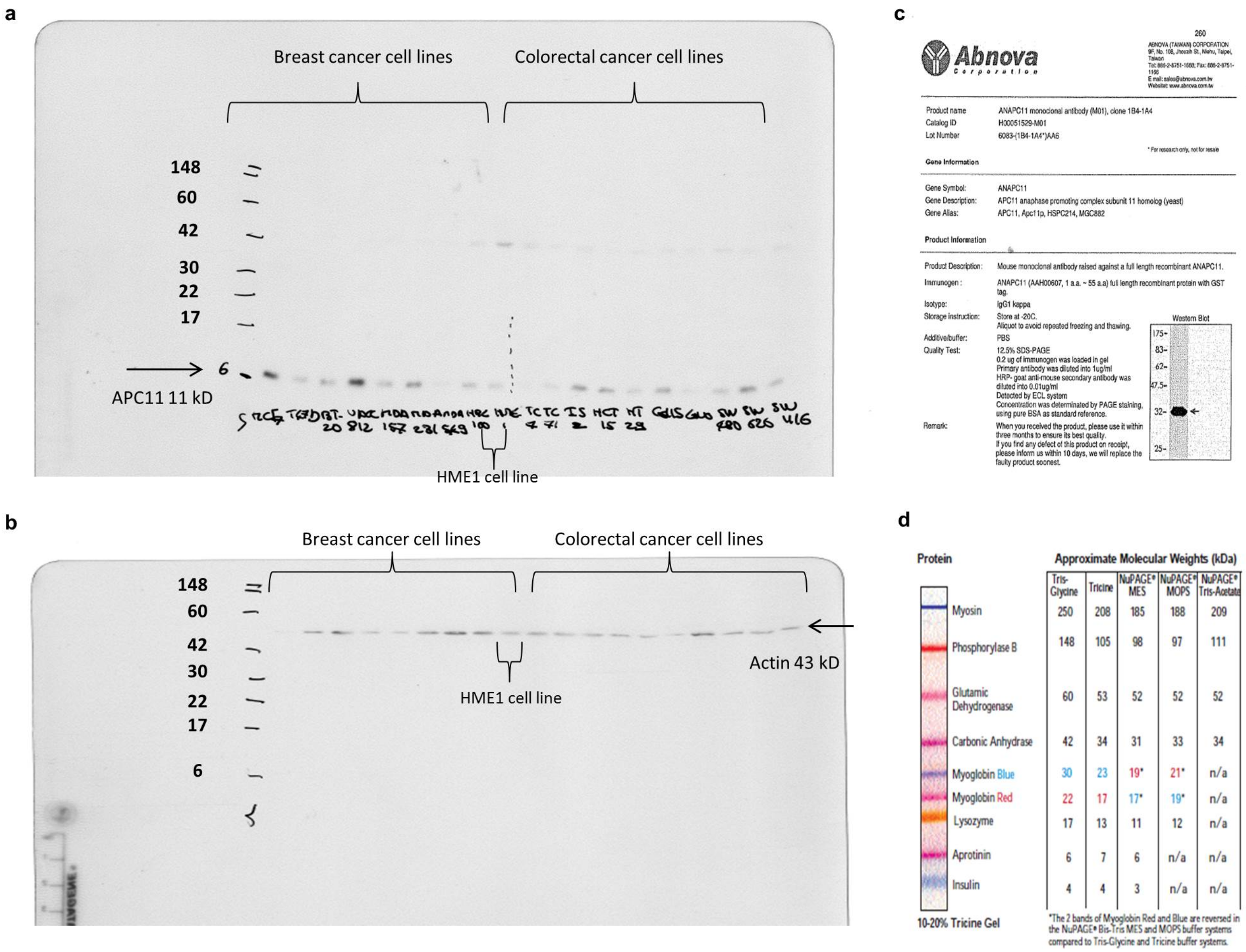

Figure S2. Uncropped Full-length gels/blots showing APC11 protein expression in a subset of colorectal and breast cancer cell lines. **(a)** Full-length gel/blot showing APC11 protein in 9 breast cancer cell lines (left-side of the blot) and in 10 colorectal cancer cell lines (right-side of the blot), molecular size markers are indicated on the blot. HME1 cell line is a non-cancerous cell line, corresponding to human immortalized mammary epithelial cells. This blot was used to realize the cropped gel/blot presented in Figure 2C. **(b)** Full-length gel/blot showing β-actin protein expression within the same gel/blot, molecular size markers are indicated on the blot. This blot was used to realize the cropped gel/blot presented in Figure 2C. **(c)** Technical data sheet showing the characteristics of the anti-APC11 monoclonal antibodies used for western-blot analyses. The band presented on the western blot corresponds to a recombinant GST-APC11 protein with an estimated size of 26 kD for the GST tag. **(d)** Technical data sheet of the MultiMark Multi-colored Standard molecular size markers used for western-blot analyses.

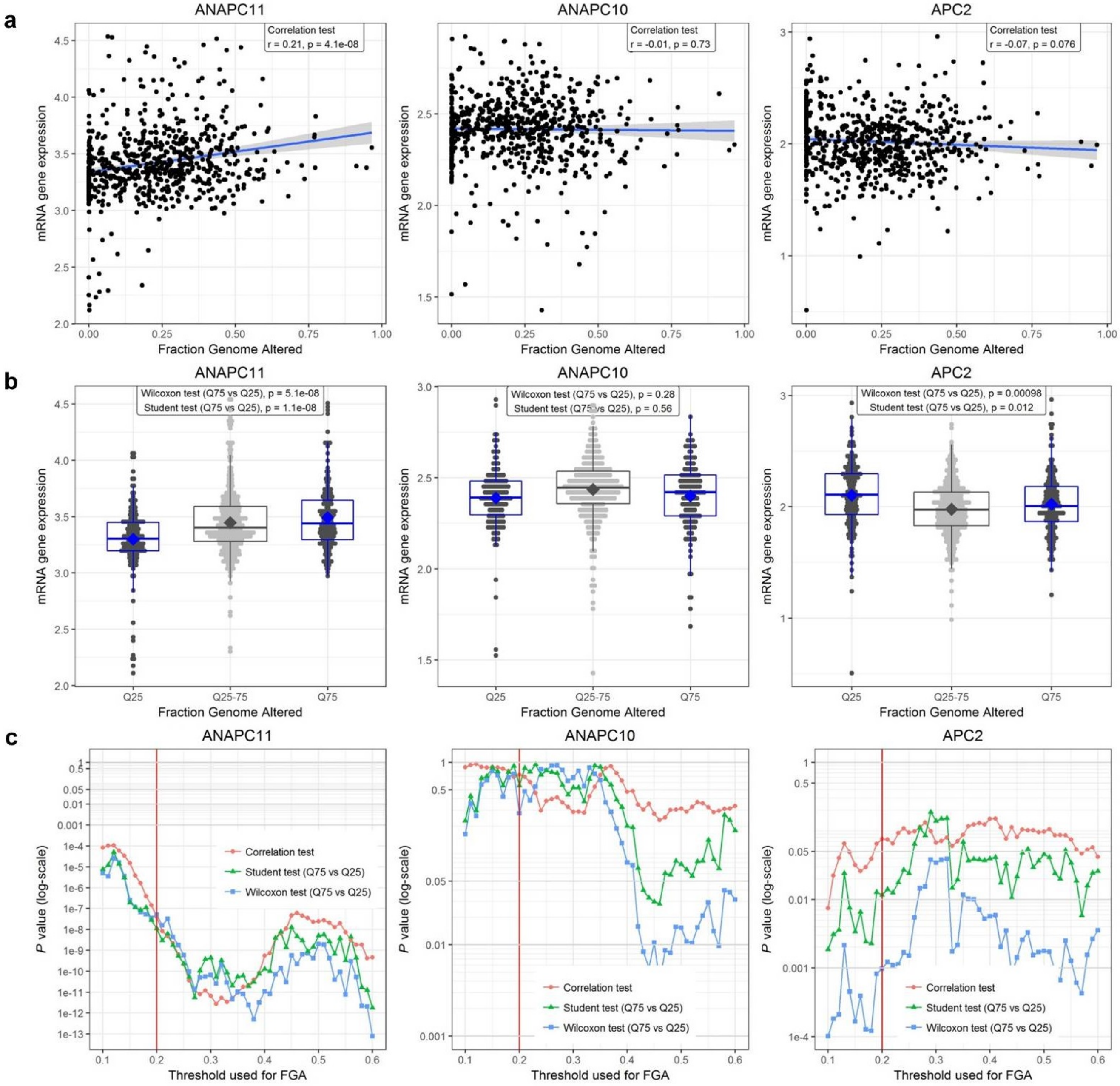

Figure S3. Analysis of the mRNA gene expression of *APC11*, *APC10* and *APC2* in primary CRC samples from TCGA repository. (a) For each gene, the top panel shows the gene expression according to the fraction of genome altered (FGA) calculated with a threshold value of 0.2. The coefficient of correlation r is displayed with the corresponding *P* value; The regression line from a linear model (blue line) and its 95% confidence interval (grey area) are also displayed. **(b)** The middle panel shows the comparison of the gene expression levels between the two extremes quartiles Q75 and Q25 of the FGA, with the non-parametric Wilcoxon test and the Student t-test. The means of the expression levels at the two extremes quartiles are indicated by blue diamonds. **(c)** The bottom panel displays the results of a sensitivity analysis for the threshold used for the FGA calculation. The p-values of the three tests (correlation, Wilcoxon and Student) are shown as a function of the threshold used for the FGA calculation. The vertical red lines indicate the value of the threshold that were used for the FGA calculations in a) and b).

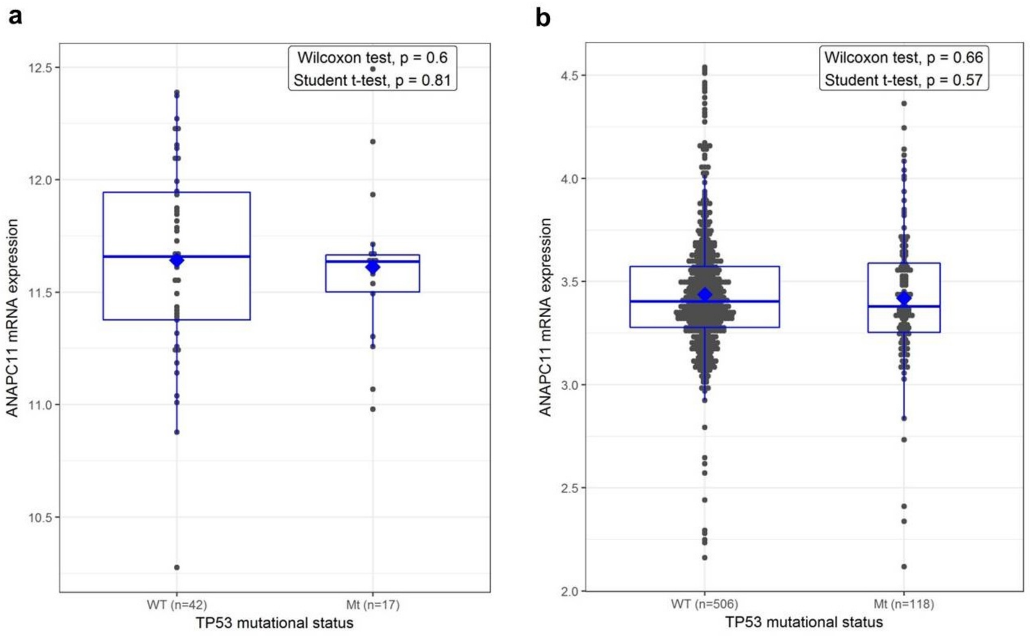

Figure S4. Analysis of the mRNA expression of *APC11* according to *TP53* mutational status. (**a**) In CRC cell lines from CCLE datasets. (**b**) In CRC samples from TCGA datasets.

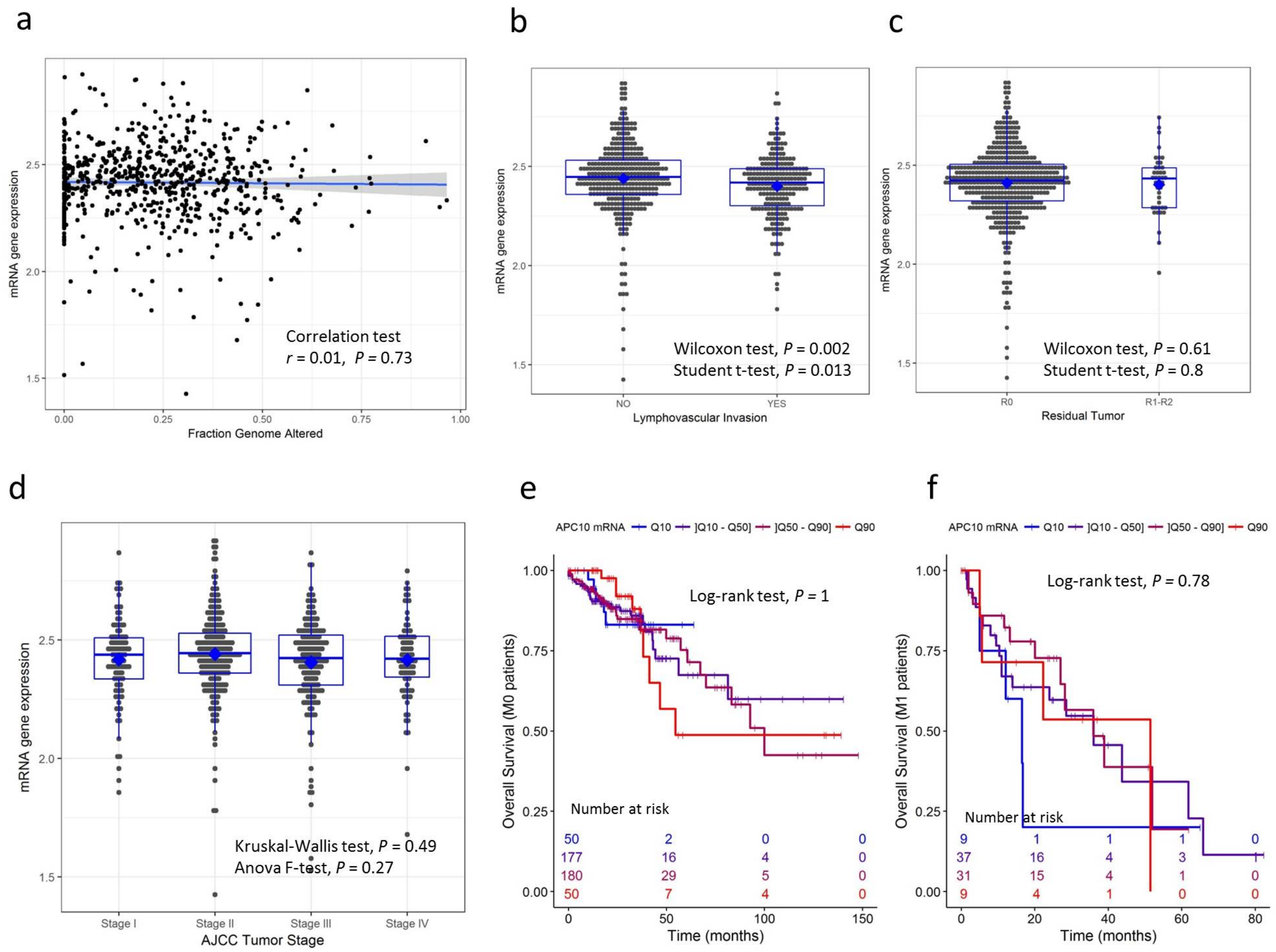

Figure S5. ***APC10*** **mRNA expression in primary CRC from the TCGA repository and statistical correlations with clinical and biological features.** Data of the TCGA READ cohort (rectum adenocarcinoma, N = 174) and the TCGA COAD cohort (colon adenocarcinoma, N = 499) were combined. **(a)** *APC10* mRNA expression according to the fraction of genome altered (FGA) calculated with a threshold value of 0.2. The coefficient of correlation r is displayed with the corresponding *P* value; The regression line from a linear model (blue line) and its 95% confidence interval (grey area) are also displayed. Panels **(b)**, **(c)** and **(d)** show respectively the *APC10* mRNA expression according to lymphovascular invasion, residual tumor status, and AJCC tumor stage. Panels **(e)** and **(f)** show the Kaplan-Meier curves of overall survival according to *APC10* mRNA expression stratified using quantiles, for patients with M0 disease (N = 457, panel e) and patients with M1 disease (N = 86, panel f).

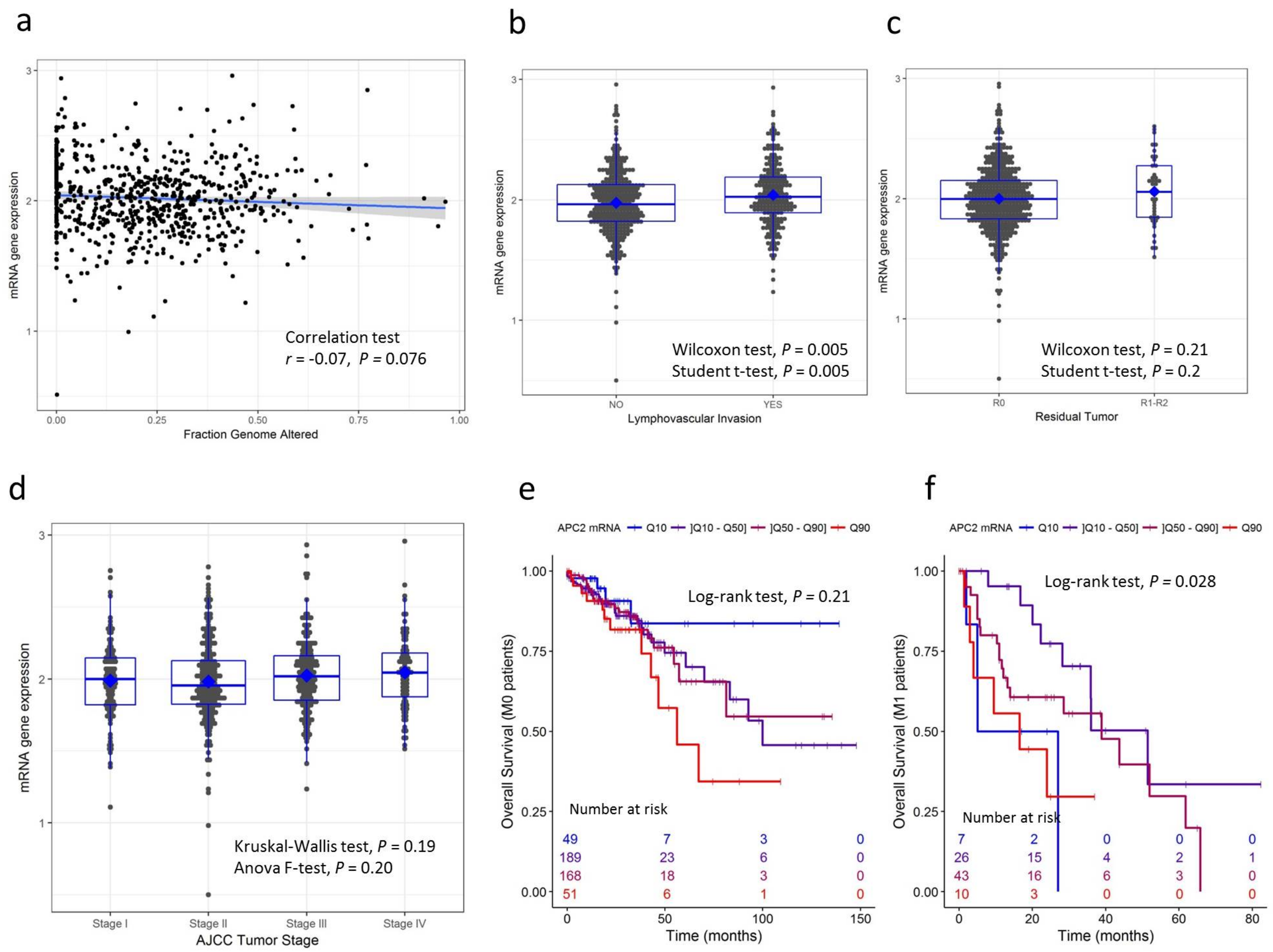

Figure S6. ***APC2* mRNA expression in primary CRC from the TCGA repository and statistical correlations with clinical and biological features.** Data of the TCGA READ cohort (rectum adenocarcinoma, N = 174) and the TCGA COAD cohort (colon adenocarcinoma, N = 499) were combined. **(a)** *APC2* mRNA expression according to the fraction of genome altered (FGA) calculated with a threshold value of 0.2. The coefficient of correlation r is displayed with the corresponding *P* value; The regression line from a linear model (blue line) and its 95% confidence interval (grey area) are also displayed. Panels **(b)**, **(c)** and **(d)** show respectively the *APC2* mRNA expression according to lymphovascular invasion, residual tumor status, and AJCC tumor stage. Panels **(e)** and **(f)** show the Kaplan-Meier curves of overall survival according to *APC2* mRNA expression stratified using quantiles, for patients with M0 disease (N = 457, panel e) and patients with M1 disease (N = 86, panel f).

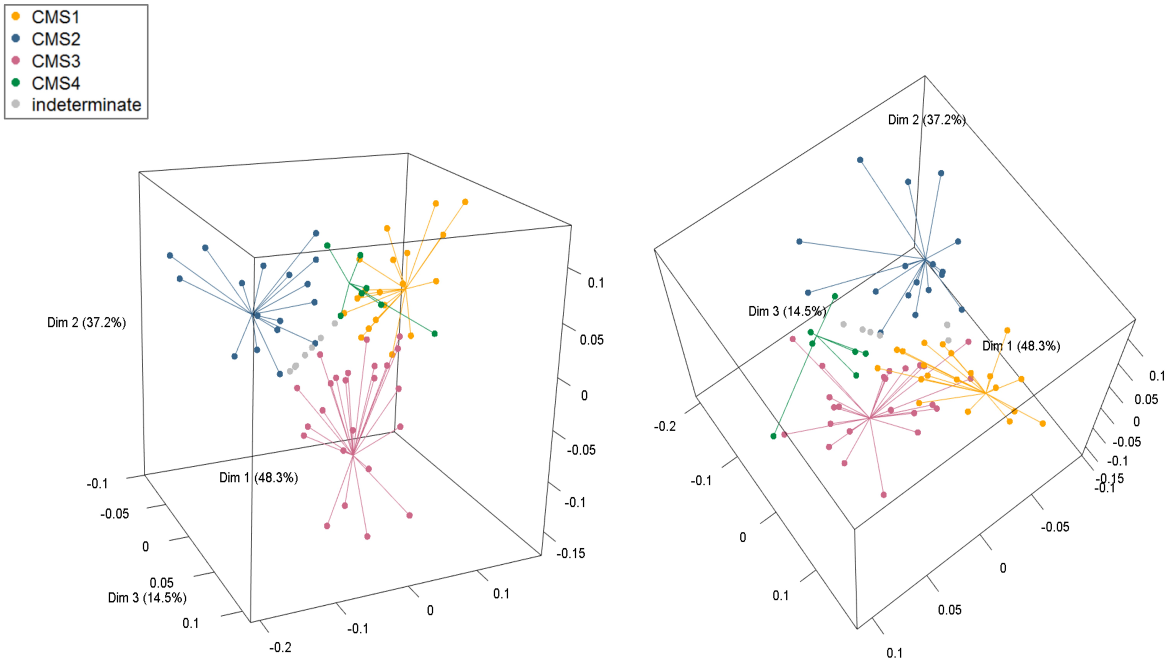

Figure S7. Two selected views from a 3-D graph showing the results of the study-specific classification for the CMS groups. This graph was obtained from a principal component analysis (PCA). Ten patients could not be classified (indeterminate, in grey) because of equal statistical similarity values with CMS2 and CMS3 groups. These 3-D views illustrate that CMS-clusters obtained with our study-specific classification are well differenciated, since CMS-clusters are well separated on the graphs.

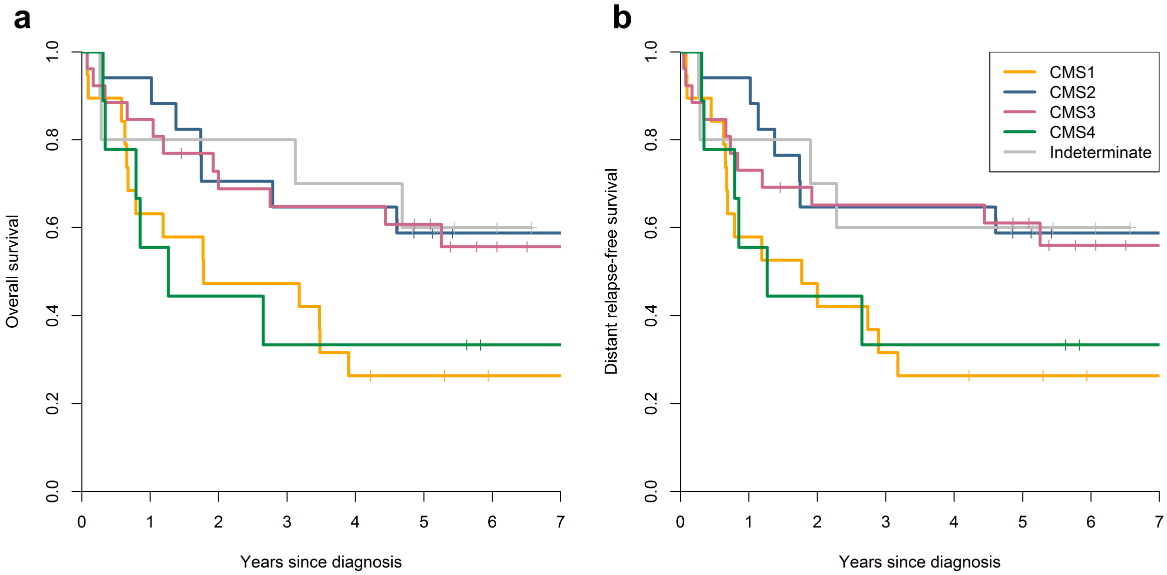

Figure S8. Kaplan-Meier curves presenting the probability of CRC patient survival (n = 81) according to the study-specific classification for the CMS groups. **(a)** Overall survival; **(b)** Distant relapse-free survival.

	APC11 expression by IHC		
Characteristics	Available (N=82, present study)	Not available (N=109)	*P*
Age at diagnosis (years)			0.26
Mean ±SD	62.58 ±13.06	64.61 ±11.57	
[Min.–Max.]	[29.41–96.74]	[33.37–89.01]	
Sex			0.66
Male	41 (50%)	58 (53%)	
Female	41 (50%)	51 (47%)	
Stage TNM			0.31
I	14 (17%)	15 (14%)	
II	26 (32%)	25 (23%)	
III	14 (17%)	29 (27%)	
IV	28 (34%)	40 (37%)	
Stage pTT4			0.96
T1	7 (9%)	9 (8%)	
T2	10 (12%)	11 (10%)	
T3	53 (65%)	74 (68%)	
T4	12 (15%)	15 (14%)	
Node involvement pN			0.37
N0	42 (52%)	46 (42%)	
N1	17 (21%)	31 (28%)	
N2	22 (27%)	32 (29%)	
Metastasis pM			0.76
M0	54 (66%)	69 (63%)	
M+	28 (34%)	40 (37%)	
Tumor residue			0.75
R0	60 (73%)	77 (71%)	
R1 and R2	22 (27%)	32 (29%)	
Tumour location			0.15
Left colon and up rectum	52 (64%)	79 (75%)	
Right and transverse colon	29 (36%)	27 (25%)	
Differentiation			0.76
Good and moderate	56 (68%)	71 (66%)	
Poor	26 (32%)	37 (34%)	
Vascular invasion			0.44
Absence	53 (66%)	62 (60%)	
Presence	27 (34%)	41 (40%)	
Stroma			0.082
Lymphoid	42 (62%)	46 (47%)	
Not lymphoid	26 (38%)	52 (53%)	
Ploidy			0.86
Diploid	23 (33%)	23 (31%)	
Aneuploid	47 (67%)	52 (69%)	
Pre-operative CEA			0.18
Normal	39 (59%)	36 (47%)	
Increased	27 (41%)	40 (53%)	

Table S1. **Clinical and histopathological characteristics at diagnosis of our series of 82 patients with colorectal cancer, according to the availability of APC11 expression.**

	CMS1	CMS2	CMS3	CMS4
E-cadherin -	1	0	0	1
E-cadherin +	0	1	1	0
BCL2+	0	0.5	0	1
BCL2-	1	0.5	1	0
p53+	0	1	0	1
p53-	1	0	1	0
MLH1+	0	1	1	1
MLH1-	1	0	0	0
MSH2+	0	1	1	1
MSH2-	1	0	0	0
KI67+	0.5	1	0.5	0
KI67-	0.5	0	0.5	1
Diploid	1	0	1	0
Aneuploid	0	1	0	1
Stroma lymphoid	1	0	0	0
stroma not lymphoid	0	1	1	1
Good or moderate differenciation	0	1	1	0
Poor differenciation	1	0	0	1
Right and transverse colon	1	0	0.5	0
Left colon and up rectum	0	1	0.5	1
Stage I-II	1	0.5	1	0
Stage III-IV	0	0.5	0	1

Table S2. Data used to obtain a study-specific classification for the CMS groups.

Study-specific CMS group	APC11 $\leq$ 50% marked cells	APC11 $>$ 50% marked cells	All
1	7 (21%)	13 (27%)	20 (24%)
2	10 (29%)	7 (15%)	17 (21%)
3	10 (29%)	16 (33%)	26 (32%)
4	2 (6%)	7 (15%)	9 (11%)
Indeterminate	5 (15%)	5 (10%)	10 (21%)

Table S3. Result of the study-specific classification for the CMS groups. Fisher's exact test: P = 0.395.

	CRC cell line characteristics							
Name	Type	MUTP53	PLOIDY	ORIGIN	DUKE	MUTAPC/C	CIN	MSI
Caco2	colon cancer	ND	aneuploid	adenocarcinoma	ND	WT	+	-
Co115	colon cancer	WT	near diploid	metastases	ND	mut apc4	-	+
Colo320	colon cancer	mutP53	aneuploid	ND	ND	WT	+	-
EB	colon cancer	mutP53	aneuploid	ND	ND	WT	+	-
FET	colon cancer	ND		ND	ND	WT	+	-
HCT116	colon cancer	WT	near diploid	adenocarcinoma	ND	WT	-	+
HCT15	colon cancer	mutP53	near diploid	adenocarcinoma	C	mut cdc16	-	+
HT29	colon cancer	mutP53	aneuploid	adenocarcinoma	ND	mut cdc23	+	-
IS1	colon cancer	mutP53	aneuploid	adenocarcinoma	C	WT	+	-
IS2	colon cancer	mutP53	aneuploid	metastases	C	WT	+	-
IS3	colon cancer	mutP53	aneuploid	metastases	C	WT	+	-
Lovo	colon cancer	WT	near diploid	metastases	C	WT	-	+
LS1034	colon cancer	mutP53	aneuploid	adenocarcinoma	C	WT	+	-
LS174T	colon cancer	WT	near diploid	adenocarcinoma	B	WT	-	+
SW1116	colon cancer	ND	aneuploid	adenocarcinoma	A	WT	+	-
SW48	colon cancer	WT	near diploid	adenocarcinoma	C	WT	-	+
SW480	colon cancer	mutP53	aneuploid	adenocarcinoma	B	mut cdc27	+	-
SW620	colon cancer	mutP53	aneuploid	metastases	C	WT	+	-
SW837	colon cancer	mutP53	aneuploid	adenocarcinoma	ND	WT	+	-
TC7	colon cancer	WT	diploid	ND	ND	WT	-	+
TC71	colon cancer	mutP53	near diploid	ND	ND	WT	-	+

Table S4. CRC cell line characteristics. Ploidy and *TP53* gene status were obtained from www.ATCC.org. APC/C genes status was determined previously (Wang *et al.* 2003).

GENE NAME	PRIMER PAIRS SEQUENCES
APC11	5’ GAGAACTGTGGCATCTGCAGGA 3’
	3’ CAGCCACTTGAGGATGCAATGC 5’
PPIB	5’ ACTTCACCAGGGGAGATGG 3’
	3’ AGCCGTTGGTGTCTTTGC 5’
β-Actin	5’ GGTCATCACCATTGGCAATG 3’
	3’ TCATACTCCTGCTTGCTGATCC 3’
PGK	5’ CTGTGGCTTCTGGCATACCT 3’
	3’ CTTGCTGCTTTCAGGACCA 5’

Table S5. Primer pairs used for relative quantification and normalization.
